# Battery-free, fully wireless neurostimulation via radiative RF power transfer in freely behaving mice

**DOI:** 10.1038/s41378-026-01439-2

**Published:** 2026-09-28

**Authors:** Kyungbin Yoo, Sehwan Park, Jimin Yang, Minseok Kim, Seungjun Lee, Giheon Kim, Namsun Chou, Hyogeun Shin, Sehoon Park

**Affiliations:** 1 School of Electronic and Electrical Engineering, Kyungpook National University, Daegu, Republic of Korea; 2 Emotion, Cognition & Behavior Research Group, Korea Brain Research Institute (KBRI), Daegu, Republic of Korea; 3 Graduate School, Daegu Gyeongbuk Institute of Science and Technology (DGIST), Daegu, Republic of Korea; 4 School of Electronics Engineering, Kyungpook National University, Daegu, Republic of Korea; 5 Brain Science and Engineering Institute, Kyungpook National University, Daegu, Republic of Korea

**Keywords:** Electrical and electronic engineering, Electronic devices

## Abstract

Wireless neurostimulators are essential for investigating causal relationships between neural circuit activity and behavior in freely behaving animals. However, most existing systems rely on onboard batteries, while near-field magnetic wireless power transfer (WPT) requires specialized environments such as large transmission coils. Radiative radio-frequency (RF) WPT enables longer transmission distances but has been considered unsuitable for freely moving conditions due to alignment sensitivity and output instability. Here, we present a battery-free, fully wireless transcranial direct current stimulation (tDCS) system based on radiative RF WPT that operates reliably in freely behaving mice. The system integrates a quasi-omnidirectional flexible printed circuit board–based monopole antenna with an RF–to–DC rectifier and a DC–DC converter, enabling robust power management despite continuous changes in position and orientation. Experiments in freely behaving mice showed that the neurostimulator maintained the regulated output voltage (~9.7 V) for the majority of the experimental period across a cage-scale environment, while transient voltage reductions occasionally occurred during abrupt posture and orientation changes. Furthermore, targeted tDCS of the M2 cortical region consistently induces circling behavior, demonstrating functional neurostimulation at the behavioral level. This work establishes the feasibility of radiative RF WPT-enabled fully wireless neurostimulation in freely behaving in vivo environments and demonstrates stable extended-duration electrical operation under continuous radiative RF powering conditions. The proposed platform provides a practical foundation for battery-free wireless neurostimulation without tethered connections or coil-embedded behavioral environments.

**Figure:**
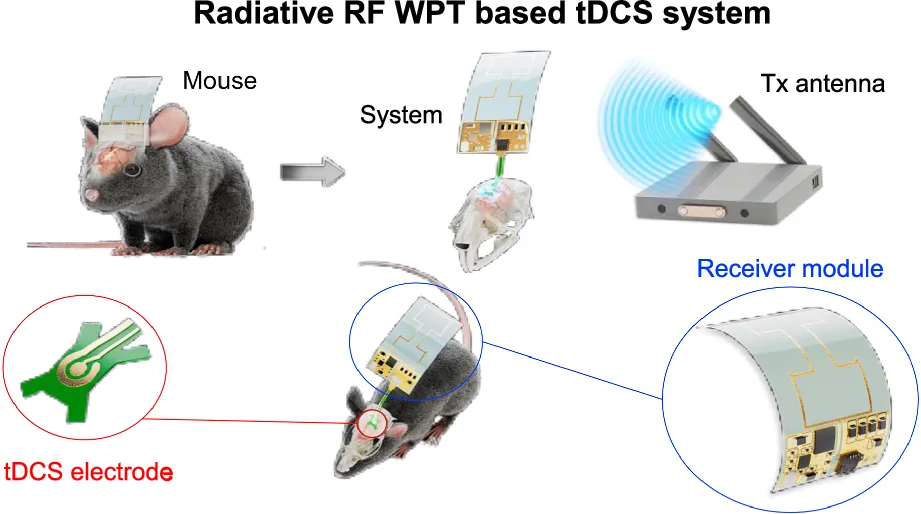

## Introduction

Neuromodulation techniques aim to alter brain function and behavior by selectively regulating the activity of targeted neural circuits and have been widely applied in both basic neuroscience research and clinical practice. Electrical stimulation^1–4^, optogenetic^5–8^, and chemical modulation^9–12^ have emerged as indispensable tools for probing neural circuit function and investigating the pathophysiology of neurological, psychiatric, and movement disorders. Accurate interpretation of neuromodulatory effects and underlying mechanisms requires experimental platforms that provide precise control over stimulation parameters while enabling direct observation of circuit-level and behavioral outcomes.

Experiments conducted in freely behaving animal models are particularly critical for establishing causal links between neural circuit modulation and behavior. Physical tethering or external constraints can substantially disrupt natural behavioral repertoires, thereby confounding the interpretation of stimulation-induced effects^13–16^. As a result, there is increasing demand for wireless neurostimulation systems that operate reliably in freely behaving conditions without imposing behavioral restrictions.

In response, a variety of wireless neurostimulators have been developed for use in mouse models, enabling untethered operation and more naturalistic behavioral assessments. However, most existing systems rely on onboard batteries as their primary power source, which fundamentally limits experimental duration and complicates long-term or repeated stimulation protocols^17–23^. Battery replacement or recharging interrupts experimental continuity and introduces additional handling stress, posing significant challenges for chronic studies.

Wireless power transfer (WPT) technologies have therefore been explored as alternatives to battery-based power supplies for neural interface systems. Near-field magnetic coupling approaches, typically operating in the MHz range, can achieve relatively high power transfer efficiency but require large transmission coils embedded in the cage floor or behavioral arena, resulting in specialized and constrained experimental environments^24–31^. In contrast, radiative radio-frequency (RF) WPT, typically operating in the GHz range and relying on propagating electromagnetic waves rather than near-field coupling, enables longer transmission distances and greater flexibility in experimental design^32–38^. Nevertheless, radiative RF WPT has been widely regarded as unsuitable for freely behaving animal experiments due to severe path loss, sensitivity to alignment, and instability of delivered power under continuous changes in position and orientation.

**Fig. 1:**
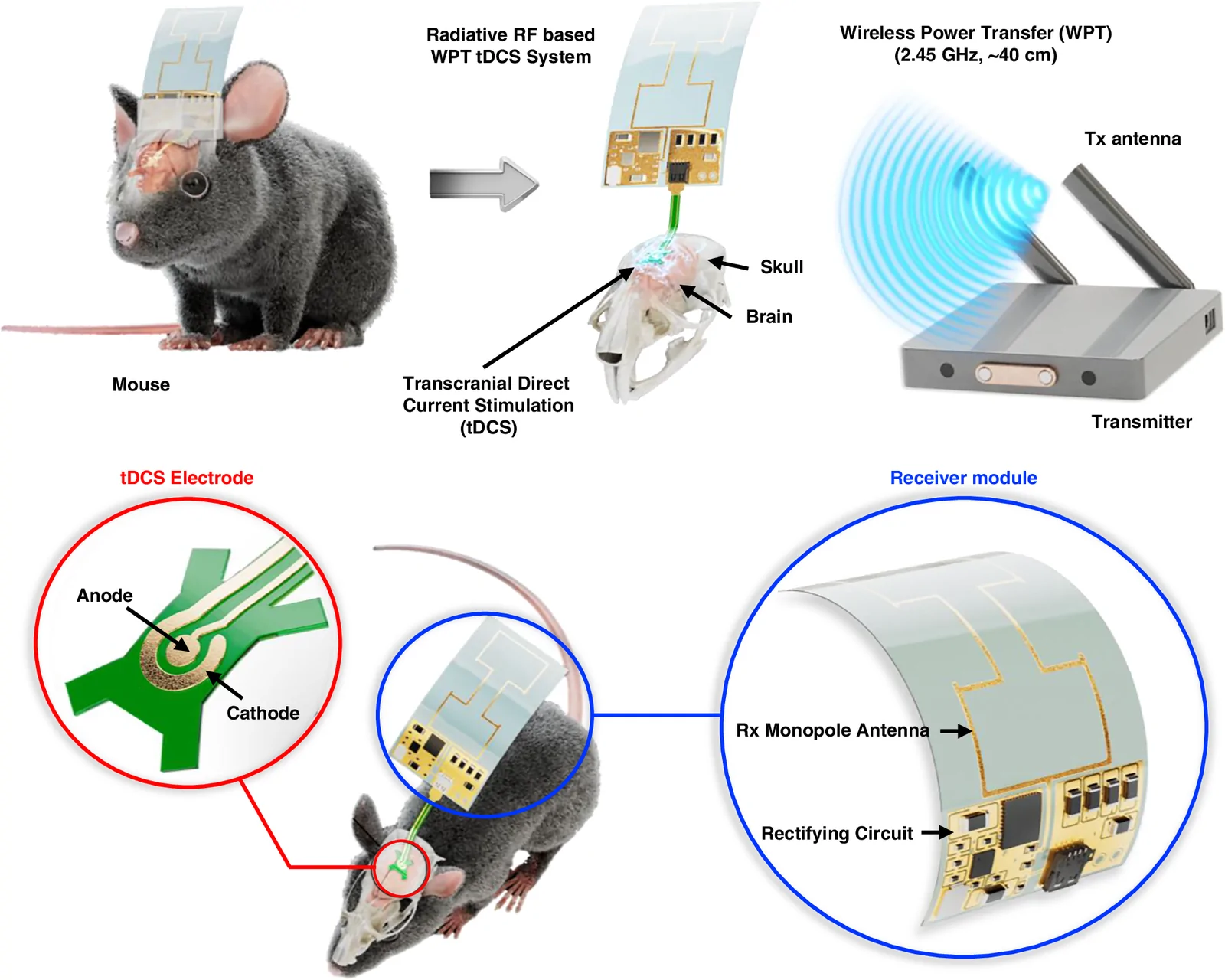
**Overview of the RF WPT–based fully wireless tDCS system for freely behaving mice.** A head-mounted tDCS neurostimulator is wirelessly powered via radiative RF transmission (~40 cm) from an external transmitter operating at 2.45 GHz. The received RF energy is captured by an integrated monopole antenna, converted into DC power, and delivered to the tDCS stimulation circuitry without onboard batteries. The generated current is applied transcranially through a flexible tDCS electrode positioned on the skull to modulate underlying brain regions

In this work, we address these limitations by presenting a battery-free, fully wireless transcranial direct current stimulation (tDCS) system based on radiative RF WPT that is specifically designed for operation in freely behaving mice (Fig. 1). Rather than attributing the challenges of radiative RF WPT solely to carrier frequency, we demonstrate that power instability primarily arises from antenna radiation characteristics and system-level integration. To address this issue, we redesign the receiver architecture by implementing a quasi-omnidirectional monopole antenna on a flexible printed circuit board (FPCB), thereby minimizing power fluctuations induced by changes in posture and orientation. A bidirectional radiation configuration that leverages the ground plane further enables dense circuit integration while maintaining sufficiently robust radiation characteristics for reliable power reception.

By integrating an RF–to–DC rectifier with a DC–DC converter, the proposed system generates a regulated high-voltage output required for tDCS across a cage-scale environment. Experimental results show that the regulated output voltage is maintained across most cage locations and during diverse behavioral states such as locomotion, rotation, and exploration in freely behaving mice, although transient voltage reductions occasionally occur during abrupt posture and orientation changes. Furthermore, when paired with an electrode array optimized for stimulation of the secondary motor cortex (M2), the system reliably induces circling behavior, demonstrating that wirelessly delivered power can elicit functional neuromodulation effects at the behavioral level. In comparison with previously reported WPT-based neurostimulators, our system eliminates the need for specially modified behavioral arenas, such as cages integrated with resonant or transmitting coils. Conventional near-field inductive systems often rely on coil-embedded enclosures or require precise alignment between the transmitting and receiving units, typically operating over only a few millimeters under well-aligned conditions (see Table S1). In contrast, the proposed radiative RF WPT approach enables wireless power delivery over an extended free-space range of ~40 cm with reduced alignment sensitivity compared with conventional near-field inductive systems. This capability enables operation across a substantially larger experimental area, thereby facilitating freely moving behavioral studies while reducing the physical and environmental constraints associated with conventional coil-based or tethered systems.

Together, these results establish the feasibility of battery-free wireless neurostimulation using radiative RF WPT in freely behaving animal models while reducing the need for coil-embedded experimental environments. The proposed platform offers a practical foundation for battery-free wireless neurostimulation and extended-duration radiative RF-powered operation, while future studies will be required to investigate chronic in vivo and repeated-session performance.

## Materials and methods

### Design and simulation of a quasi-omnidirectional FPCB monopole antenna

The antenna used in this study was fabricated using an FPCB process (Dawon Flex Co., Ltd., South Korea) to enable a conformal, head-mounted platform suitable for wearable applications. Polyimide (PI) was selected as the substrate material, and the antenna structure was implemented using a two-layer FPCB stack-up. Copper patterns on the top and bottom layers served as the radiating element and ground plane, respectively.

The top layer consisted of a coverlay (PI, 25 μm), adhesive (30 μm), copper plating (Cu, 15 μm), electrodeposited copper (Cu/ED, 12 μm), and adhesive (10 μm). The bottom layer comprised PI (15 μm), adhesive (10 μm), Cu/ED (12 μm), copper plating (Cu, 15 μm), adhesive (30 μm), and a coverlay (PI, 25 μm). To improve mechanical rigidity and handling stability, a stiffener (K/D, 165 μm) was attached to the bottom of the structure, resulting in a total thickness of 364 μm (Fig. S1).

The antenna pattern was defined on the top copper layer. To satisfy both miniaturization and radiation requirements for radiative RF WPT, a meandered monopole geometry was employed. This configuration increases the effective electrical length within a compact footprint and was optimized to achieve dual-band resonance at the 2.45 and 5.8 GHz ISM bands.

In addition to resonance tuning, the radiation pattern was engineered to exhibit quasi-omnidirectional characteristics. This design objective was adopted to mitigate sensitivity to rotational motion and orientation variation, which are unavoidable in freely behaving conditions.

**Fig. 2:**
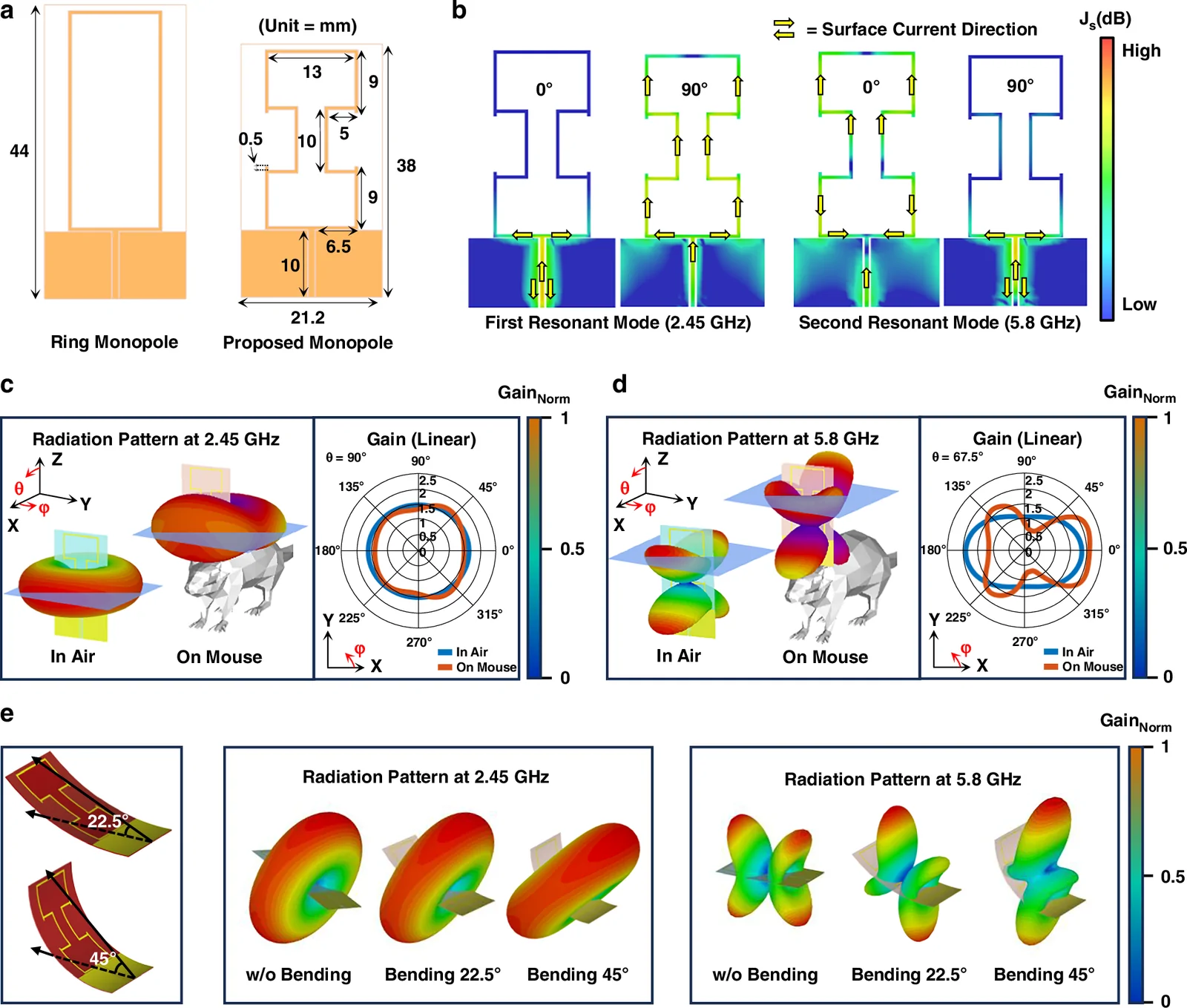
**Design, simulation, and characterization of the quasi-omnidirectional FPCB monopole antenna.** **a** Ring monopole antenna (left) and geometric layout and dimensions of the meandered FPCB-based monopole antenna (right). **b** Simulated surface current distributions (*J*_s_) at 2.45 and 5.8 GHz for excitation phases of 0° and 90°. The surface current distribution is plotted in dB(A m^−1^) to visualize the relative current intensity over a wide dynamic range. **c** Simulated three-dimensional radiation patterns of the proposed antenna at 2.45 and 5.8 GHz in free space and **d** when mounted on a mouse model (*ε* = 45). The linear gain distributions are compared on planes parallel to the x–y plane at selected elevation angles (*θ* = 90° at 2.45 GHz and *θ* = 67.5° at 5.8 GHz) to evaluate the influence of biological loading on angular radiation characteristics. **e** Radiation robustness under mechanical deformation, evaluated through simulated radiation patterns and normalized gain at 2.45 and 5.8 GHz under no bending, 22.5°, and 45° bending conditions. *Gain_Norm_: normalized linear gain, where Gain_Norm_ = Gain/Gain_Max_, Gain_Max_ = 2

Key geometric parameters, including line width, spacing, and segment length, were optimized through parametric sweeps using CST and HFSS electromagnetic simulation tools. The final optimized antenna dimensions are shown in Fig. 2a.

To ensure robust performance under mechanical deformation, radiation characteristics under bending conditions were simulated and compared with those of the flat configuration, enabling simultaneous evaluation of mechanical flexibility and electromagnetic stability.

### Design of RF WPT power management circuit

To ensure efficient delivery of the received RF power to the rectification stage, the entire receiving chain—including the antenna, voltage doubler, and DC–DC converter—was considered as a unified loaded system during the impedance-matching design. The voltage doubler was modeled using the manufacturer-provided Schottky diode SPICE model, incorporating intrinsic device parameters and package parasitics specified in the datasheet. In addition, the diode’s large-signal behavior under 2.45 GHz excitation was experimentally characterized to refine the nonlinear model parameters. Specifically, the RF input power at the doubler input port was incrementally increased while monitoring the rectified DC output voltage, and the onset of high-field nonlinear behavior was identified when the output voltage exceeded ~8 V. Based on this large-signal RF characterization, the effective breakdown-related parameters in the nonlinear model were adjusted to reproduce the experimentally observed rectifier behavior under practical radiative RF WPT conditions. The Zener diode was represented using a measurement-based nonlinear model calibrated to its measured current–voltage (I–V) characteristics (Fig. S2). The DC–DC converter was modeled using its measured steady-state equivalent input resistance (Fig. S3). For tDCS operation, the stimulation load was further approximated as a 100 kΩ equivalent resistance.

At 2.45 GHz, the free-space wavelength is ~122 mm, corresponding to ~65–68 mm on a PI substrate. As a result, even millimeter-scale interconnects and parasitic elements can significantly influence the input impedance and matching network. In the FPCB structure employed in this study, the two metal layers are separated by ~35 μm, resulting in an ultra-thin configuration. Such small interlayer spacing significantly enhances parasitic capacitance and near-field electromagnetic coupling between adjacent conductive regions. At 2.45 GHz, these parasitic interactions not only modify the intrinsic antenna input impedance but also introduce additional reactive loading around the matching network components. As a result, the designed matching network may deviate from its intended impedance transformation characteristics under integrated conditions.

To suppress these unintended parasitic effects and restore stable matching behavior, selective ground-plane trimming was implemented in the CPW monopole region to enhance electromagnetic isolation between the matching network components and adjacent conductive elements. Although this trimming introduces slight modifications to the antenna input impedance and minor asymmetry in the radiation pattern (Fig. S4), it effectively disrupts unintended current return paths along the ground plane that were electromagnetically coupled to the DC routing and matching components. This modification represents a deliberate system-level design optimization aimed at minimizing input power reflection while maintaining robust RF–to–DC conversion performance.

Accordingly, an ADS–based circuit simulation model was developed, incorporating the antenna input impedance; parasitic capacitances and inductances extracted using CST associated with the FPCB layout; two 10 pF capacitors (QLCD250Q100G1GV001T, Johanson Technology); Schottky diode (SMS7621-005LF, Skyworks) and package parasitics; a Zener diode (1N4731A, Lumimax); the equivalent input resistance of the DC–DC converter; and three 10 μF capacitors (CL05A106MP5NUNC, Samsung Electro-Mechanics). The input impedance at the antenna port was extracted from the CST simulation (see Table S2), and conjugate matching was achieved using a series RF matching network comprising 1 nH (LQP03HQ1N0B02D, Murata) and 4.5 nH (LQP03HQ4N5H02D, Murata) inductors.

For RF–to–DC conversion, a Greinacher-type voltage doubler employing low–forward-voltage Schottky diodes was implemented to maximize conversion efficiency under the limited input power typical of radiative RF WPT. The doubler capacitor value (10 pF) was selected to balance RF impedance requirements with the input characteristics of the subsequent DC–DC converter.

A DC–DC converter (MAX17291, Analog Devices) was used to regulate the rectified voltage, providing conversion efficiencies exceeding 70% at a load current of 100 μA over an input voltage range of 1.8–5.5 V. The output voltage was configured using an external passive feedback network in accordance with the manufacturer’s design guidelines, incorporating three 10 μF capacitors, an 8.2 μH inductor (PSTMAA4030-8R2MB, PROD Tech), 402 kΩ resistor (CRCW0402402KFKED, VISHAY), 2.49 MΩ resistor (CRCW04022M49FKED, VISHAY) and five 4.7 μF Capacitors (GRM035R60J475ME15D, Murata Electronics). To protect the converter from overvoltage conditions arising from excessive received RF power, a Zener diode clamp with a breakdown voltage of 4.3 V was integrated at the converter input.

### RF power transmission and electrical characterization

An EXG analog signal generator (N5171B, Keysight) was used to generate the RF signal, which was subsequently amplified using a dual-band 2.45/5.8 GHz power amplifier (ACASOM). A meandered monopole antenna supplied with the amplifier served as the transmitting antenna.

The received RF power (P_in_) was derived from the link budget equation (Table S3),

1 $${{{\rm{P}}}}_{{{\mathrm{in}}}}\left({{\mathrm{dBm}}}\right)={{{{\rm{P}}}}}_{{{\mathrm{SG}}}}-{{{\rm{L}}}}_{{{\mathrm{Cable}}}}+{{{{\rm{G}}}}}_{{{\mathrm{PA}}}}+{{{{\rm{G}}}}}_{{{\rm{t}}}}-{{{\rm{L}}}}_{{{\mathrm{FSPL}}}}+{{{{\rm{G}}}}}_{{{\rm{r}}}}$$

which accounts for signal generator output power, cable loss, power amplifier gain, measured transmitter antenna gain, free-space path loss calculated according to the Friis transmission equation and the simulated receiver antenna gain on full system without components (excluding receiver-to-rectifier mismatch effects),

2 $${{{\rm{L}}}}_{{{\mathrm{FSPL}}}}({{{\mathrm{dB}}}})=20{{{\mathrm{log}}}}_{10}\left(\frac{4\pi R}{\lambda }\right)$$

To evaluate DC output characteristics, voltage and current were measured using a 100 kΩ resistive load $({R}_{{load}})$, corresponding to the validated operating conditions of the tDCS stimulation circuit. A high-impedance digital multimeter was connected across the load, and the output current was calculated from the measured voltage (*V*_out,meas_) and known resistance.

The system-level efficiency was defined as the ratio of the load power to the received RF power. For the measurements, the output power (P_out,meas_) was calculated as

3 $${{{\rm{P}}}}_{{{{\mathrm{{out}}}}},{{{\mathrm{meas}}}}}=\frac{{V}_{{out},{meas}}^{2}\,}{{R}_{{{\mathrm{load}}}}}$$

and the corresponding efficiency (η_meas_) was calculated by

4 $${\upeta }_{{{\mathrm{meas}}}}=\frac{{P}_{{out},{meas}}\,}{{P}_{{in}}}.$$

In the simulation, the receiver input power was swept over a defined range, and the DC–DC converter was modeled using a measurement-based steady-state equivalent input resistance. The simulated input power (P_in,DCDC,sim_) to the converter was obtained from the rectifier simulation, and the output power (P_out,sim_) was calculated by applying the independently measured DC–DC conversion efficiency (η_DCDC,meas_ ≒ 72%), i.e.,

5 $${{{\rm{P}}}}_{{{\mathrm{out}}},{{\mathrm{sim}}}}={\upeta }_{{{\mathrm{DCDC}}},{{\mathrm{meas}}}} \cdot {{{\rm{P}}}}_{{{\mathrm{in}}},{{\mathrm{DCDC}}},{{\mathrm{sim}}}}.$$

The simulated efficiency (η_sim_) was then defined as

6 $${\upeta }_{{{\mathrm{sim}}}}=\frac{{P}_{{out},{sim}}\,}{{P}_{{in}}}.$$

### SAR simulation and thermal measurement

To evaluate RF exposure safety under radiative wireless power transfer conditions, specific absorption rate (SAR) simulations were performed using CST Microwave Studio. A homogeneous dielectric mouse model was employed to estimate localized RF absorption. The entire mouse model was assigned brain-equivalent dielectric properties at 2.45 GHz based on the IT’IS Foundation database, with a relative permittivity of 44.8 and an electrical conductivity of 2.1 S m^−1^ ^39^. Although this simplified model does not account for tissue-specific dielectric heterogeneity or anatomical complexity, it provides a practical approximation for localized RF exposure assessment.

The SAR simulation environment is illustrated in Fig. S5. The transmitting antenna was positioned above the mouse model using the same geometric configuration employed in the freely behaving experiments. To evaluate localized RF exposure under the worst-case transmitter-to-receiver configuration, SAR simulations were performed with the transmitting and receiving antennas positioned in the closest possible configuration. The transmitter input power was set to 4 W, corresponding to the maximum RF power used during the behavioral experiments. Peak spatial-average SAR was calculated using the IEEE/IEC 62704-1-compliant averaging algorithm with a 0.1 g spatial-averaging mass^40^.

To experimentally evaluate potential RF-induced thermal effects during wireless power transmission, scalp temperature measurements were performed in four freely behaving mice using an infrared thermal camera. Temperature recordings were continuously acquired for 10 min under RF OFF and RF ON conditions. During RF ON measurements, continuous 2.45 GHz RF power transmission at 4 W was applied using the same transmitter configuration employed in behavioral experiments. Thermal images were acquired every 6 s, and the maximum scalp temperature was extracted from each frame for analysis.

### Experimental setup for distance and orientation dependence

Experiments were conducted inside an acrylic cage with dimensions of 40 × 40 × 15 cm^3^. A three-dimensional coordinate system was defined with the cage center as the origin. The transmitting antenna was fixed at the top center of the cage using a custom support structure and oriented vertically along the *z* axis to maintain consistent polarization alignment.

The receiving module, mounted on a head-mounted crown identical to that used in behavioral experiments, was attached to a 3D-printed plastic dummy mouse model and oriented vertically along the *z* axis. This vertical configuration preserves polarization alignment with the transmitting antenna and minimizes susceptibility to orientation-induced power fluctuations under freely behaving conditions.

To further evaluate the influence of mounting orientation, additional simulations were performed with the receiving antenna oriented horizontally above the mouse head (Fig. S6). In this configuration, the radiation pattern exhibited increased asymmetry and reduced effective gain toward the transmitter direction due to polarization misalignment and enhanced interaction with the biological loading structure. Although the horizontally mounted receiver exhibited a higher intrinsic peak gain, its main radiation direction was misaligned with the dominant radiation direction of the vertically oriented transmitting antenna, resulting in reduced effective power reception. Moreover, under freely behaving conditions, a horizontally oriented receiving antenna inevitably experiences time-varying polarization mismatch due to instantaneous body rotation and head tilt, leading to fluctuations in received power.

To quantitatively evaluate spatial dependence, the transmitter–receiver distance (*r*), azimuthal angle (*φ*), and tilt angle (*θ*) were defined. The distance r was measured between the phase centers of the transmitting and receiving antennas. The azimuthal angle *φ* represented rotation about the vertical axis, simulating free-body rotation, while the tilt angle θ represented head tilt from the vertically aligned condition and was varied from 0° to 100°.

For each (*r*, *φ*, *θ*) configuration, the DC output voltage after the rectification and power management stages was recorded. The transmitter power required to achieve a target DC output voltage was calculated. The results were visualized using heat maps to quantify spatial sensitivity and to analyze the dependence of WPT performance on distance and orientation.

### Fabrication of the CE-tDCS Electrode

The CE-tDCS electrodes were fabricated using a standard FPCB process based on protocols validated in previous studies^41–43^. Briefly, a 12-µm-thick copper layer was laminated onto a PI substrate and patterned via wet etching to define the electrode and interconnection structures. An insulating layer was subsequently applied using photoimageable solder resist ink, after which the stimulation sites and contact pad regions for FPC connector interfacing were selectively opened using photolithography. The exposed copper surfaces were coated with electroless nickel/gold (Ni/Au) to improve electrical conductivity and ensure biocompatibility.

The electrode architecture consisted of a centrally positioned anode enclosed by a surrounding cathode. The anode was designed as a circular electrode with a diameter of 0.35 mm, while the cathode formed a concentric ring structure surrounding the central anode, with inner and outer radii of 0.3 mm and 0.5 mm, respectively. The anode–cathode separation was fixed at 0.125 mm.

To enhance mechanical robustness and ensure reliable electrical interfacing with the FPC connector, a 0.3-mm-thick stiffener was attached to the terminal region of the electrode. The final electrode outline was defined by laser cutting, resulting in a total electrode thickness of ~35 µm. The fabricated CE-tDCS electrodes were electrically connected to the RF WPT–based tDCS system via a 4-pin FFC/FPC connector (503480-0400, Molex).

To provide stable mechanical support for both the skull-mounted tDCS electrode and the receiver module, a custom-designed crown structure was fabricated using a 3D printer (K1 Max, Creality). This crown was mounted above the electrode assembly to maintain mechanical stability of the integrated system under freely behaving conditions.

### Animals

All animal experiments were approved by the Institutional Animal Care and Use Committee (IACUC) of the Korea Brain Research Institute (KBRI, Daegu, Republic of Korea) and were conducted in accordance with the institute’s Animal Care and Use Guidelines. All in vivo experiments were conducted using male C57BL/6 mice (≥10 weeks old, body weight ≥25 g). The number of animals used for each experiment was as follows: output voltage measurement (*n* = 4), temperature measurement during repeated RF power ON/OFF operation (*n* = 4), open field test (OFT) before and after system mounting (*n* = 3), OFT during repeated RF power ON/OFF operation (*n* = 4), and M2 cortex stimulation experiments (*n* = 4). Animals were individually housed in open-top cages (38 × 23 × 19.5 cm^3^) under controlled temperature and humidity conditions and maintained on a 12 h light/12 h dark cycle with ad libitum access to food and water.

### Surgical procedure

During surgery, mice were secured in an automated stereotaxic frame (71000 Automated Stereotaxic Instrument, RWD Life Science, USA). Anesthesia was induced and maintained using an isoflurane vaporizer (Screw Filler Cagemount, Medical Supplies & Services, UK), with 4% isoflurane used for induction and 1.5–2% during surgical procedures.

After stable anesthesia was achieved, the scalp was incised to expose the skull. For more effective stimulation, the drill was mounted on an automated stereotaxic frame (71000 Automated Stereotaxic Instrument, RWD Life Science, USA), and the skull over the target stimulation region—the secondary motor cortex (M2)—was carefully thinned. In addition, to ensure mechanical stability during subsequent in vivo experiments, a stainless steel anchoring screw was fixed to the skull on the left side of the target stimulation region (M2). Based on the Paxinos and Franklin mouse brain atlas^44^, the relative positions between bregma and the reference holes located at the center of the tDCS electrode were used to accurately target the bilateral M2 regions (mediolateral (ML): ±0.55 mm; anteroposterior (AP): +1.0 mm).

The tDCS electrode was carefully aligned and placed directly onto the skull surface. A thin layer of PMMA-based dental cement (Vertex Self-Curing, Vertex Dental B.V., The Netherlands) was applied to ensure intimate contact between the electrode and the skull. Additional dental cement was subsequently applied around the electrode and anchoring screws to reinforce mechanical fixation and long-term stability.

To securely mount the radiative RF WPT-based system, a custom-designed crown structure fabricated using a 3D printer (K1 Max, Creality, China) was positioned over the electrode and aligned with the anchoring screws. Additional dental cement was applied between the skull and the crown structure to ensure long-term structural stability. Following surgery, mice were returned to their home cages and allowed to recover for at least 1 week prior to subsequent experiments.

### Output voltage stability during freely moving conditions

To evaluate the operational stability of the radiative RF WPT-based tDCS system under freely behaving conditions, oscilloscope-based output voltage measurements were performed while mice moved freely within a standard home-cage environment (38 × 23 × 19.5 cm^3^).

The transmitting antenna was positioned above the cage and connected to a power amplifier (ACASOM, China), while a radio-frequency signal generator (N5171B, Keysight, Korea) delivered a continuous 2.45 GHz RF signal. The transmitting antenna was fixed in a vertical orientation relative to the cage floor, allowing natural variations in transmitter–receiver distance and azimuthal orientation to arise from spontaneous animal movements.

To measure the output voltage, thin measurement wires were soldered to the capacitor output node of the tDCS electrode and to the system ground. These signals were connected to a mixed-signal oscilloscope (Analog Discovery 3, Digilent, USA) and recorded continuously throughout the experiment. Simultaneously, freely behaving mice were video-recorded using a smartphone camera positioned outside the cage to enable synchronized behavioral observation. Each recording session lasted 6 minutes. The acquired voltage waveforms were exported in .csv format and visualized using GraphPad Prism (GraphPad Software, USA).

Behavioral videos were subsequently analyzed offline and manually categorized into static behaviors (resting, sniffing, feeding) and dynamic behaviors (moving, grooming, digging). The timing of each behavioral event was aligned with the voltage recordings to qualitatively assess the relationship between behavioral state transitions and output voltage fluctuations under freely behaving conditions.

### Current-density simulation under voltage conditions

To evaluate the influence of transient voltage fluctuations on effective tDCS delivery during freely behaving operation, current-density simulations were performed using Sim4Life Lite (ZMT, Switzerland).

The simulation domain consisted of layered skull and brain tissue models with electrical properties defined based on the IT’IS database. The skull and brain tissues were assigned conductivities of 0.02 S m^−1^ and 0.04 S m^−1^, respectively. Dirichlet boundary conditions were applied to the electrode surfaces, while Neumann boundary conditions were used for the outer simulation boundaries.

The electrode configuration consisted of a central anode surrounded by a concentric cathode structure corresponding to the CE-tDCS geometry used in the experimental system. To evaluate the effect of transient voltage reductions observed during freely behaving operation, simulations were performed using the maximum and minimum 1-second averaged output voltages experimentally measured during wireless operation, corresponding to 9.7 V and 5.65 V, respectively.

### Current measurement under realistic electrode-loading conditions

To experimentally evaluate the delivered stimulation current under realistic electrode-loading conditions, a skull–brain phantom was constructed to emulate the electrical loading encountered during transcranial stimulation. The equivalent resistance of the mouse skull was estimated based on the electrical parameters used in the previous current-density simulations. Using the skull conductivity (0.02 S m^−1^), skull thickness (0.2 mm), and CE-tDCS anode diameter (0.35 mm), the skull-mimicking resistance was approximated using the cylindrical resistance model,

7 $${{{\rm{R}}}}_{{{\mathrm{skull}}}}=\frac{L}{\sigma A}$$

where *L* is the skull thickness, *σ* is the skull conductivity, and *A* is the cross-sectional area of the anode electrode. Since this analytical model assumes one-dimensional current flow through a cylindrical conductor rather than three-dimensional current spreading within the skull, it was used as a simplified approximation for selecting the equivalent loading resistance. Based on this calculation, a 100 kΩ resistor (R_skull_) was employed to represent the electrical resistance of the skull.

To emulate the conductive properties of the brain tissue, a 0.9% agarose gel prepared in 1× PBS was used^45^. The agarose gel was prepared by dissolving 0.3 g of agarose powder in 30 g of 1× PBS. To ensure complete dissolution, the mixture was heated in a microwave oven for 10 s and stirred, and this procedure was repeated six times. After the solution became completely transparent, it was allowed to solidify at room temperature for 30 min.

The skull-mimicking resistor (*R*_skull_) and agarose gel were connected in series to construct the skull–brain phantom. The proposed wireless stimulation system was then operated under repeated RF power ON/OFF conditions using the same transmitter configuration as that employed in the wireless stimulation experiments. To monitor the RF output voltage during the experiment, lead wires were soldered to the output terminals of the system and connected to a digital multimeter, enabling continuous real-time measurement of the output voltage throughout the experiment. The delivered current through the skull–brain phantom was measured using a SourceMeter (Keithley 2612B, Keithley Instruments, USA) connected in series with the phantom load and continuously recorded throughout the experiment.

### Open field test

To evaluate whether the total weight of the WPT–based tDCS system (~1.8 g) affected spontaneous locomotor behavior, an OFT was conducted. Each mouse underwent two OFT sessions: one prior to surgery and one after implantation of the wireless tDCS system, using the same animals in both conditions.

During the pre-surgery session, each mouse was placed in an open-top acrylic arena (40 × 40 × 40 cm^3^) and allowed to freely explore the environment for 20 min. Locomotor behavior was continuously recorded using a camera mounted above the arena. Ambient lighting and background noise conditions were kept constant to minimize external influences on behavior. Following surgical implantation, mice were allowed a minimum recovery and habituation period of 1 week. The OFT was then repeated under identical experimental conditions with the radiative RF WPT-based tDCS system mounted.

To further evaluate the potential behavioral effects of continuous RF power transmission, additional OFT experiments were performed under sequential RF OFF and RF ON conditions using the wireless system without tDCS electrode attachment. During these experiments, 4 W RF power corresponding to the maximum RF input condition evaluated in the present study was continuously transmitted. Each mouse was allowed to freely explore the same acrylic arena (40 × 40 × 40 cm^3^) during sequential 3 min RF OFF and 3 min RF ON sessions while locomotor behavior was continuously recorded using an overhead camera.

Recorded behavioral videos were analyzed using idTracker software to extract quantitative locomotion metrics, including total distance traveled and average movement speed^46^. These parameters were compared between pre- and post-implantation conditions to assess whether system mounting induced significant alterations in spontaneous motor behavior.

### Circling behavior test under M2 stimulation

To functionally validate the neuromodulatory capability of the radiative RF WPT-based tDCS system, a behavioral assay based on circling behavior was performed. Each experimental session lasted 6 min and consisted of a 3-min baseline (OFF) period without stimulation followed by a 3-min stimulation (ON) period during which electrical stimulation was applied.

During the experiment, a power amplifier (ACASOM, China) was connected to the transmitting antenna positioned above the cage in a fixed vertical orientation. A radio-frequency signal generator (N5171B, Keysight, Korea) delivered a continuous 2.45 GHz RF signal. The head-mounted tDCS electrode remained coupled to the receiver module, enabling electrical stimulation to be delivered to the right secondary motor cortex (M2) upon RF power reception.

Animal behavior was continuously recorded using a camera mounted above the cage throughout the session. Behavioral analysis was performed offline using MATLAB (R2024a, MathWorks, USA). A circling event was defined as a complete 360° rotation around the animal’s vertical body axis performed in place.

To minimize subjective bias, circling events were independently counted by two observers blinded to the stimulation condition. The number of circling events during the baseline and stimulation periods was compared and visualized using GraphPad Prism (GraphPad Software, USA).

## Results and discussion

### System overview

This study presents a radiative RF WPT–based system for battery-free tDCS in freely behaving animals (Fig. 1). The overall system comprises an RF transmitter (Tx), a free-space RF power transmission path, and a head-mounted receiver (Rx) affixed to the mouse skull. The transmitter emits RF power in the 2.45 GHz ISM band, which propagates through free space and is captured by the receiver antenna. The received RF energy is converted into DC and directly supplied to the tDCS stimulation circuitry, enabling fully wireless neuromodulation without onboard batteries or tethered connections.

The receiver module is implemented as an integrated system consisting of an FPCB–based monopole antenna, an RF–to–DC rectifier, a DC–DC converter, and a tDCS stimulation module. This architecture is optimized at the system level to support stable wireless power reception for the majority of freely behaving operation despite continuous variations associated with natural animal behavior. The head-mounted configuration explicitly accounts for dynamic changes in distance and relative orientation between the transmitter and receiver under freely behaving conditions.

A central design principle of the proposed system is that the limitations traditionally associated with radiative RF WPT—namely alignment sensitivity and power fluctuations—can be mitigated through antenna radiation characteristics and system-level integration rather than carrier frequency alone. Accordingly, the receiver antenna is engineered to exhibit quasi-omnidirectional radiation characteristics, thereby minimizing abrupt reductions in received power during locomotion, rotation, and exploratory behaviors. In addition, the antenna ground plane is leveraged as an integration platform for the power management circuitry, enabling dense and compact system integration while maintaining sufficiently robust radiation characteristics for reliable power reception.

Captured RF power is first converted into DC voltage using a voltage-doubler–based rectifier and subsequently regulated by a DC–DC converter to generate the regulated high-voltage output required for tDCS operation. Through this power processing chain, the system delivers sufficient electrical power to drive the stimulation circuitry across a cage-scale environment. As a result, the proposed platform enables fully wireless, battery-free tDCS in freely behaving animal experiments without the need for large transmission coils or coil-embedded experimental environments.

Although the present implementation still requires an overhead transmitting antenna and a vertically mounted receiver structure for radiative coupling, the system eliminates physical tethering and substantially reduces the environmental and mechanical constraints commonly associated with coil-embedded near-field wireless power transfer systems.

### Design, simulation, and characterization of a quasi-omnidirectional FPCB monopole antenna

In experimental settings involving freely behaving animals, the relative distance, orientation, and polarization alignment between the transmitter and receiver vary continuously over time. Under these conditions, receiver antennas optimized for a specific direction are highly susceptible to power degradation. Therefore, a receiving antenna with radiation characteristics that are robust to orientation changes is essential. To address this requirement, we designed an FPCB–based monopole antenna with quasi-omnidirectional radiation characteristics to reduce sensitivity to posture and orientation changes during freely behaving operation^47^.

Figure 2a illustrates the ring monopole antenna and the geometry of the proposed antenna. The antenna is implemented as a meandered monopole structure on a PI substrate, which increases the effective electrical length within a compact footprint and achieves resonance in the 2.45 GHz ISM band^48^. In addition, the structure is designed to support resonance at 5.8 GHz, enabling compatibility with commonly used Wi-Fi frequency bands and extending applicability to multiband RF wireless power transfer scenarios^47^.

Electromagnetic simulations were first performed on a 50 Ω reference configuration, whose feed structure differs from that of the fully integrated receiver, to evaluate the intrinsic resonant and radiation characteristics of the proposed antenna structure independent of system-level loading effects.

Figure 2b shows the simulated surface current distributions at 2.45 and 5.8 GHz at instantaneous excitation phases of 0° and 90°, demonstrating that the antenna supports resonant modes at both frequencies, with the effective electrical length appropriately tuned to satisfy the resonance condition at each band.

We simulated three-dimensional radiation patterns of the proposed antenna at 2.45 and 5.8 GHz under free-space conditions and when mounted on a mouse model (*ε* = 45) (Fig. 2c, d). The corresponding linear gain distributions are evaluated on planes parallel to the x–y plane at selected elevation angles (*θ* = 90° at 2.45 GHz and θ = 67.5° at 5.8 GHz) to assess the influence of biological loading on angular radiation characteristics. In addition, we confirmed radiation intensity distributions observed from six principal viewing directions (top, bottom, front, back, left, and right) (Fig. S7). The antenna exhibits a nearly uniform azimuthal radiation profile at both frequency bands without pronounced radiation nulls. Importantly, when vertically mounted above the mouse model (*ε* = 45), the overall radiation pattern is largely preserved, indicating that dielectric loading from skin, skull, and brain tissue does not significantly degrade antenna performance. To assess robustness against mechanical deformation in a head-mounted configuration, antenna bending angles of 22.5° and 45° were simulated. No significant degradation in radiation pattern or gain is observed under these conditions, confirming stable performance under deformation induced by animal motion (Fig. 2e, Table S4).

The RF performance of the fabricated 50 Ω matched antenna was experimentally validated through reflection coefficient (S11) measurements. The antenna exhibits reflection losses of −8.96 dB at 2.45 GHz and −9.43 dB at 5.8 GHz, indicating adequate impedance matching for RF power reception (Fig. S8). These measurements are in good agreement with simulation results, supporting the validity of the proposed standalone antenna design.

In summary, the proposed FPCB-based monopole antenna demonstrates quasi-omnidirectional radiation characteristics that are robust to mechanical deformation and biological loading. Although the radiation characterization was performed using a 50 Ω reference-fed configuration, additional simulations confirm that the antenna on a fully integrated system—with its distinct feed and loading conditions—preserves the overall quasi-omnidirectional radiation characteristics without significant qualitative deviation (Fig. S4).

These comparative results demonstrate that vertical mounting is not merely a practical choice but a necessary design condition to preserve polarization alignment with the transmitting antenna and to maintain stable radiative coupling under freely behaving conditions. The vertically oriented configuration was therefore adopted throughout subsequent system-level and in vivo experiments.

Although the proposed antenna supports dual-band operation at both 2.45 and 5.8 GHz, all in vivo wireless neurostimulation experiments in this study were conducted using the 2.45 GHz band. Compared with 5.8 GHz operation, the 2.45 GHz band provides lower free-space propagation loss and a larger effective power-delivery region within the cage-scale experimental environment. These characteristics improve tolerance against posture- and position-dependent fluctuations associated with freely behaving conditions and enable more stable wireless power delivery during practical freely behaving conditions involving continuous posture and orientation changes. Therefore, the 2.45 GHz band was selected in the present study to prioritize robust wireless power transfer under practical freely behaving experimental conditions.

### Fabrication and electrical characterization of the RF WPT-based tDCS system

**Fig. 3:**
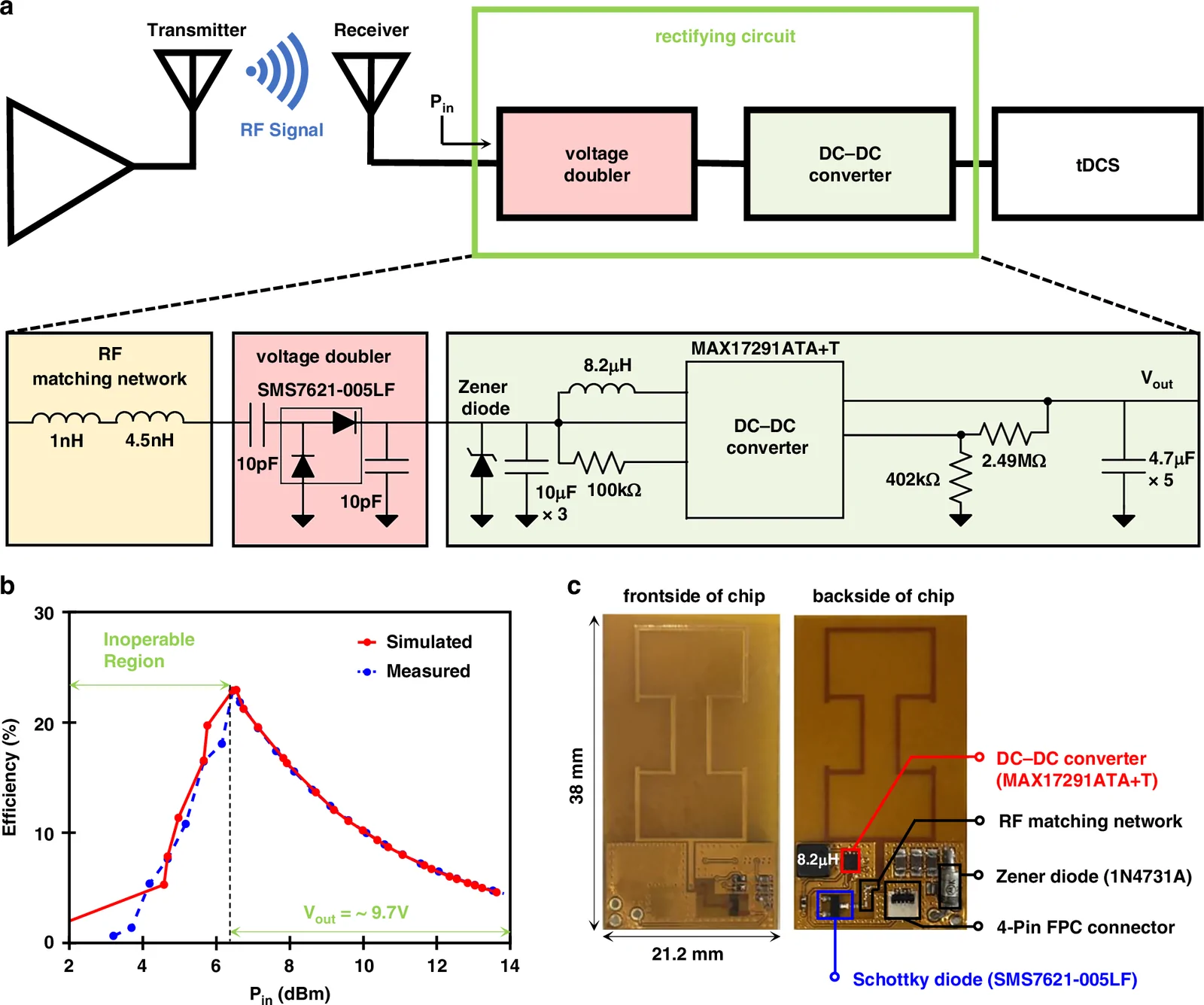
**Fabrication and electrical characterization of the radiative RF WPT–based tDCS system.** **a** Circuit schematic of the receiver module, consisting of an RF impedance matching network, a Greinacher-type voltage doubler, a Zener diode for input voltage protection, and a DC–DC converter for generating a stable high-voltage output required for tDCS operation. **b** RF–to–DC conversion efficiency as a function of input RF power, comparing simulated and experimentally measured results at a dummy mouse. **c** Photographs of the fabricated receiver module showing the front side (left) and back side (right) of the module. Key functional components—including the Schottky diode, Zener diode, RF matching network, DC–DC converter, and 4-pin FPC connector for interfacing with the tDCS electrode—are annotated

The proposed wireless tDCS system is designed to support battery-free neurostimulation under radiative RF WPT conditions. The rectifying circuit comprises an impedance-matching network following the antenna, an RF–to–DC conversion stage based on a voltage doubler, a Zener diode for input voltage protection, and a DC–DC converter that generates the regulated high-voltage output required for tDCS operation (Fig. 3a).

RF power captured by the receiving antenna is first delivered to the matching network to maximize power transfer to downstream circuitry. The matched RF signal is then rectified and voltage-doubled using a Greinacher-type voltage doubler. Because the received RF power in radiative environments can vary substantially with distance and relative orientation, excessive input voltages may arise under certain conditions. To protect downstream components from overvoltage, a Zener diode is integrated at the output of the voltage doubler to clamp the input voltage of the DC–DC converter within a safe operating range. The regulated input is subsequently converted by a DC–DC converter into a regulated output voltage suitable for driving the tDCS stimulation circuitry.

To quantitatively evaluate the performance of the power management circuit, RF power transmission experiments were conducted with the receiver module mounted inside a crown structure positioned on a 3D-printed plastic dummy mouse model, using a signal generator and a transmitting antenna operating at 2.45 GHz. The resulting DC output characteristics at the receiver were experimentally measured under a 100 kΩ load, corresponding to realistic system operating conditions^17^. Figure 3b compares the simulated and measured RF–to–DC conversion efficiency as a function of the estimated input RF power (*P*_in_). The experimental results show good qualitative agreement with simulation and demonstrate the generation of a regulated DC output when the input RF power exceeds the minimum power required for voltage regulation.

The system is designed to produce a regulated output voltage of ~9.7 V at the output of the DC–DC converter, which is sufficient for tDCS operation. Experimentally, the output voltage remained within the range of 9.63–9.88 V acros s the tested RF input power levels, indicating effective voltage regulation. Importantly, after the minimum required RF input power is reached, the output voltage remains relatively constant despite increases in input power, indicating that the Zener diode and DC–DC converter effectively suppress RF power fluctuations inherent to radiative WPT.

Figure 3c shows a photograph of the fabricated receiver module. All functional components, including the antenna pattern, RF matching network, voltage doubler, Zener diode, DC–DC converter, and an FPC connector for interfacing with the tDCS electrode, are integrated on a single FPCB. This fully integrated architecture enables battery-free wireless operation within a compact integrated head-mounted platform.

Collectively, these results experimentally demonstrate that the proposed radiative RF WPT–based tDCS system can support regulated high-voltage DC generation under radiative wireless power transfer conditions. This electrical characterization establishes the foundation for subsequent evaluations under freely behaving conditions and for in vivo neurostimulation experiments.

### Output voltage stability under distance and orientation variations

**Fig. 4:**
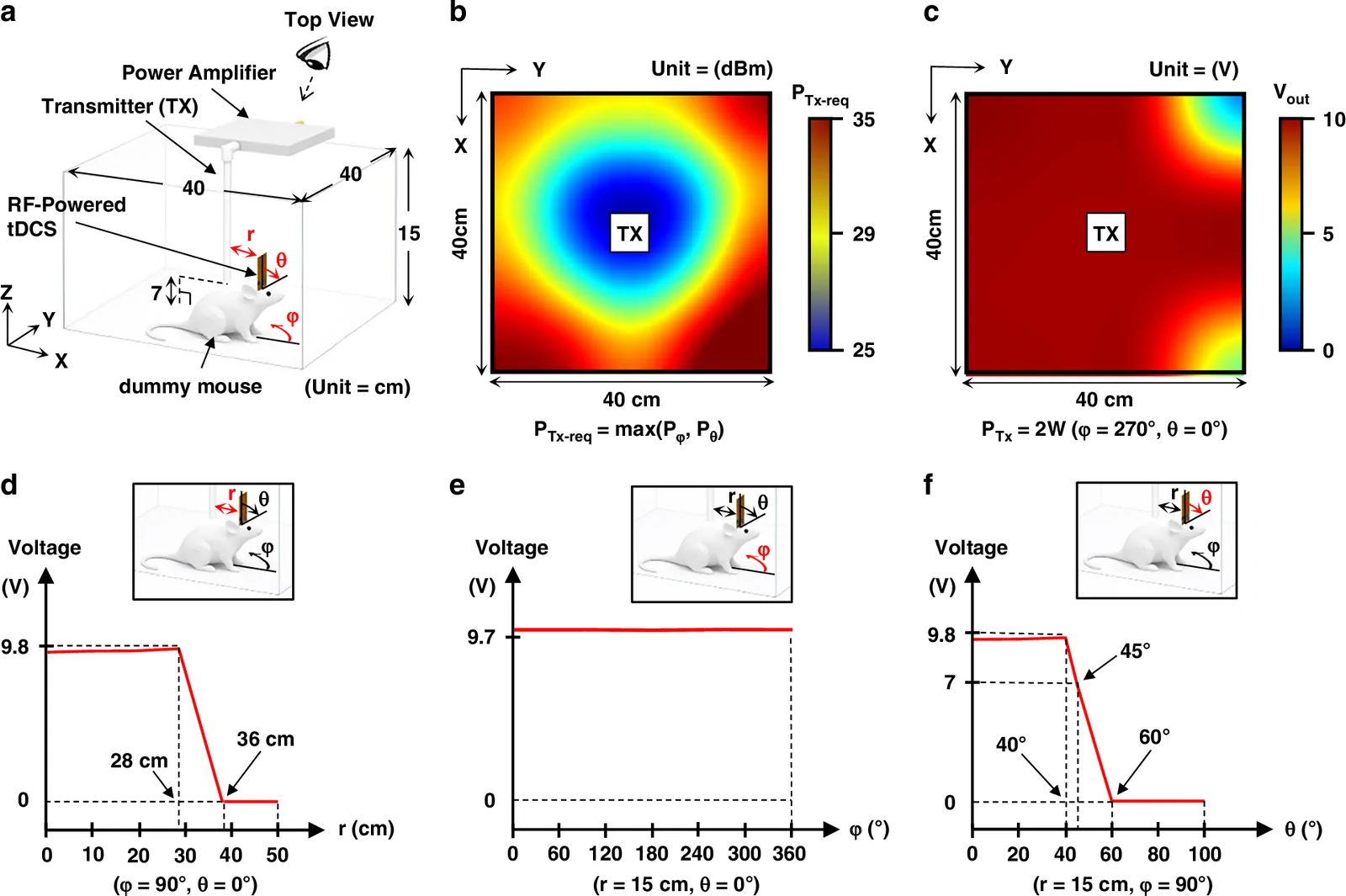
**Output voltage stability of the radiative RF WPT-based tDCS system under distance and orientation variations.** **a** Experimental configuration illustrating the spatial relationship between the RF transmitter and the head-mounted receiver in a cage-scale environment. The relative horizontal distance (*r*), azimuthal angle (*φ*), and tilt angle (*θ*) of the receiver module were systematically varied to emulate positional and postural changes during freely behaving conditions. **b** Spatial heat map of the required transmitter power (P_Tx-req_) for the radiative RF WPT-based system within a 40 × 40 × 15 cm³ experimental volume. For each spatial location, P_Tx-req_ represents the transmitter power necessary to maintain the target DC output voltage while accounting for variations in both azimuthal angle (*φ*) and tilt angle (*θ*). All values are expressed in dBm. **c** Measured DC output voltage distribution across the x–y plane when the transmitter output power was fixed at 2 W (*φ* = 270°, *θ* = 0°). **d** Output voltage as a function of horizontal transmitter–receiver separation distance (*r*) under 4 W RF transmission, showing a stable regulated output (~9.7 V) up to ~28 cm (*φ* = 90°, *θ* = 0°). **e** Output voltage as a function of azimuthal angle (*φ*) under 4 W RF transmission at a fixed distance (*r* = 15 cm, *θ* = 0°), indicating minimal sensitivity to rotational orientation over the full 360° range. **f** Output voltage as a function of receiver tilt angle (*θ*) under 4 W RF transmission at a fixed distance (*r* = 15 cm, *φ* = 90°), where stable regulation is maintained up to ~40°

To assess whether the proposed radiative RF WPT–based tDCS system operates reliably under practical experimental conditions, we systematically characterized output voltage stability with respect to variations in transmitter–receiver distance and relative orientation (Fig. 4a). These variations emulate realistic changes in spatial position and posture encountered during freely behaving animal experiments.

In the experimental setup, the transmitting antenna was fixed above the cage, while the receiving module was positioned below in a head-mounted configuration (Fig. S9). The transmitter–receiver geometry was varied to reflect both horizontal displacement in the x–y plane and vertical separation along the z-axis. In addition, the azimuthal angle (*φ*) and tilt angle (*θ*) of the receiving module were independently adjusted to evaluate their effects on system performance.

To quantify spatial power delivery characteristics, we first determined the transmitter output power required to achieve a target DC output voltage of ~9.7 V at the receiver across different positions. Figure 4b presents a heat map of the required transmitter power (*P*_Tx-req_), showing that regulated operation is achieved with relatively low transmitter power across most regions directly beneath the transmitter. This result indicates that the combination of the quasi-omnidirectional receiving antenna and the power management circuitry provides sufficient tolerance to positional variations.

To further examine large-area spatial robustness, additional experiments were conducted in an expanded acrylic enclosure measuring 80 × 80 × 15 cm³ (Fig. S10). Even within this enlarged test volume, regulated operation was maintained over a substantial region directly beneath the transmitter, confirming that the system is not limited to small-scale cage geometries.

To assess system performance under a controlled and conservative power condition, the transmitter output power was then fixed at 2 W, which is lower than the 4 W used in the in vivo experiments, and the resulting DC output voltage was measured as a function of receiver position. The output voltage remains close to 9.7 V across the majority of the cage area, with only modest degradation observed near the periphery (Fig. 4c). Although Fig. 4c illustrates measurements at a representative orientation, additional φ-sweep and *θ*-sweep experiments were performed to further validate angular robustness (Fig. S11). These supplementary measurements show that the output voltage remained close to 9.7 V within the tested angular ranges, including azimuthal rotation over 0°–360° and tilt angles up to ~40°, beyond which voltage degradation became more pronounced.

Distance-dependent behavior was further examined by incrementally increasing the horizontal separation (*r*) between the transmitter and receiver while monitoring the output voltage (Fig. 4d). The output voltage remains nearly constant up to a distance of ~28 cm (*φ* = 90°, *θ* = 0°), beyond which a rapid voltage drop is observed. This trend is consistent with the expected attenuation characteristics of radiative RF propagation^49^.

The influence of receiver orientation was then evaluated. When the azimuthal angle (*φ*) was varied from 0° to 360° (*r* = 15 cm, θ = 0°), the output voltage remained stable across the entire range (Fig. 4e), indicating effective mitigation of polarization mismatch due to the vertical alignment of the transmitting and receiving antennas. In contrast, increasing the receiver tilt angle (*θ*) resulted in stable output voltage up to ~40° (*r* = 15 cm, *φ* = 90°), beyond which a gradual reduction was observed (Fig. 4f).

The asymmetry observed in the measured voltage is consistent with electromagnetic simulations performed using an antenna on a fully integrated system that includes the DC measurement leads. During electrical characterization, these DC leads are necessarily connected to enable voltage monitoring. The simulations indicate that the added leads electromagnetically couple to the antenna ground plane and feed transition, inducing common-mode currents that perturb the intrinsic radiation characteristics. In contrast, during actual wireless stimulation experiments (Fabrication of the CE-tDCS electrode), the system operates without the external measurement wiring used for voltage monitoring, thereby eliminating this unintended parasitic radiation path. Comparative simulations conducted with and without the DC leads further confirm that the radiation distortion is substantially suppressed in the absence of the measurement wiring (Fig. S12). These results collectively demonstrate that the observed directional bias originates from parasitic effects introduced by the voltage-monitoring leads rather than from the inherent antenna geometry or system architecture.

Overall, these controlled distance- and orientation-dependent measurements demonstrate that the proposed radiative RF WPT–based tDCS system maintains wireless power delivery across practical spatial and postural variations despite occasional voltage degradation under large tilt-angle conditions. Although voltage degradation becomes more pronounced under large tilt-angle conditions, the observed fluctuations were transient in nature.

### SAR and thermal safety evaluation

To evaluate the RF exposure safety of the proposed radiative RF WPT system, SAR simulations and in vivo thermal measurements were performed under the experimental conditions.

SAR simulations were conducted under the worst-case transmitter-to-receiver configuration to evaluate localized RF absorption. Under this worst-case exposure condition, the simulated peak spatial-average SAR was ~16 W kg^−1^.

Because the reported SAR value was calculated using the IEEE/IEC 62704-1 0.1 g spatial-averaging procedure, it is not directly comparable with the localized SAR limits specified using a 10 g averaging mass in the ICNIRP and IEEE human exposure guidelines. Furthermore, the SAR simulation was performed using a homogeneous dielectric mouse model with brain-equivalent dielectric properties assigned throughout the model. Therefore, the simulated SAR value should be interpreted as an approximate estimate of localized RF absorption rather than a direct measure of tissue-specific RF exposure.

To experimentally evaluate potential RF-induced heating, scalp temperature measurements were performed during continuous RF transmission in freely behaving mice (Fig. S13 and Movie S1). Across four independent animals, only minimal temperature variation was observed during RF exposure, with an average temperature increase of ~0.03 °C over the 10 min measurement period. Representative thermal imaging recordings further confirmed the absence of noticeable localized heating during wireless power transmission (Movie S1).

Collectively, although the SAR simulation provides an approximate assessment of localized RF absorption under the worst-case exposure condition, the in vivo thermal measurements provide direct experimental evidence that the proposed radiative RF WPT system does not produce measurable RF-induced thermal effects during freely behaving experiments. Therefore, the thermal measurements provide the primary experimental evidence supporting the RF safety of the proposed system.

### Output voltage stability during freely behaving conditions

**Fig. 5:**
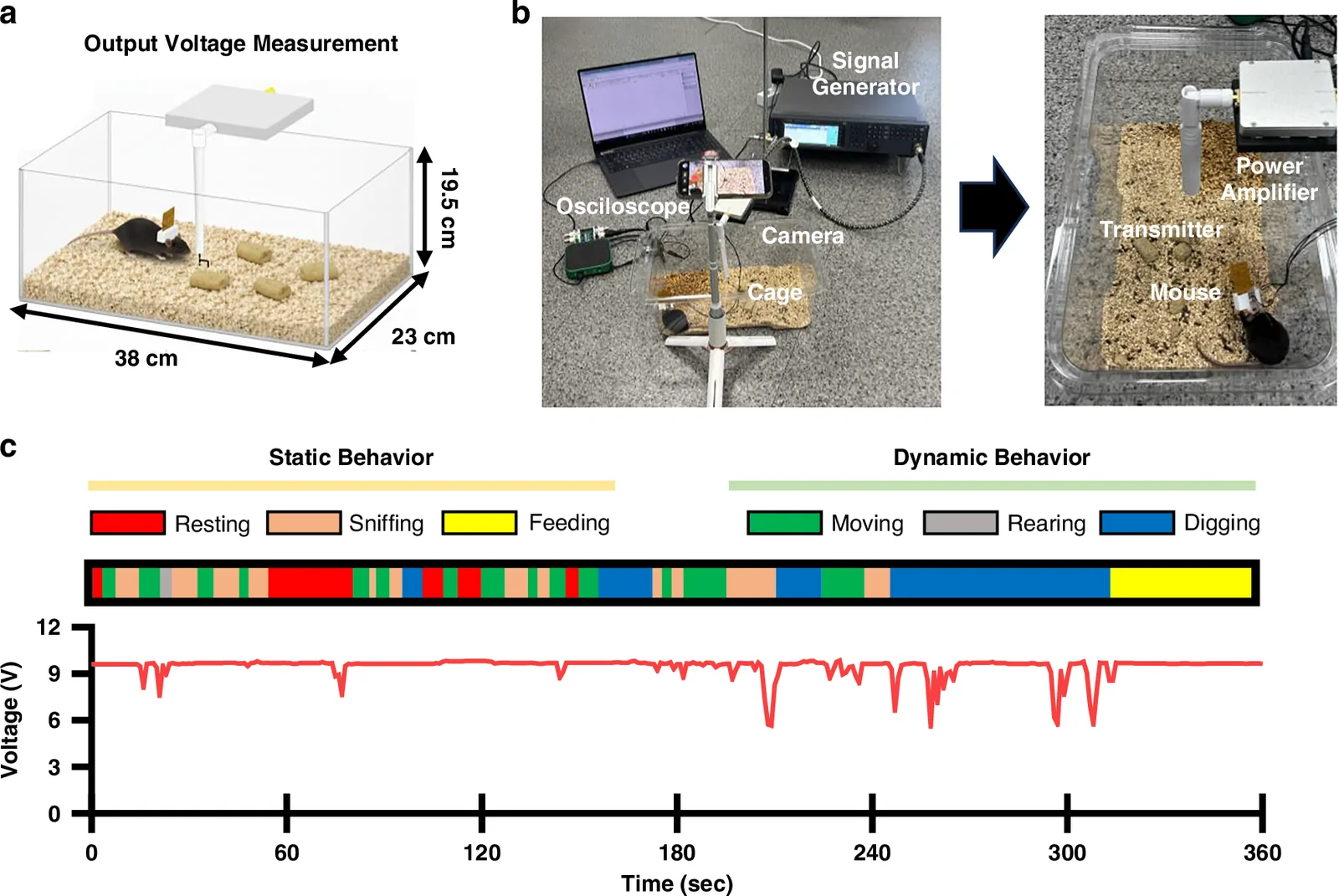
**Output voltage stability of the RF WPT neurostimulator during freely behaving conditions.** **a** Schematic of the home-cage experimental setup for continuous output voltage monitoring. The head-mounted RF WPT neurostimulator was operated in a cage (38 × 23 × 19.5 cm³), and the output voltage was recorded for 6 min under freely behaving conditions. **b** Photographs of the experimental setup, including the signal generator, power amplifier, overhead camera, and oscilloscope used for simultaneous voltage recording and behavioral video acquisition. **c** Representative time-aligned behavioral annotations (top) and corresponding output voltage trace (bottom). Behaviors are categorized as static and dynamic, showing that the output voltage remains near the regulated level (~9.7 V) during natural posture and position changes

To evaluate the power delivery stability of the proposed radiative RF WPT–based tDCS system under realistic freely behaving conditions, the output voltage was continuously monitored while mice moved freely within a home-cage environment. Experiments were conducted in a cage measuring 38 × 23 × 19.5 cm^3^, with the receiving module mounted on the head of the mouse throughout the measurement period (Fig. 5a). The transmitting antenna was fixed above the cage, enabling continuous wireless RF power delivery to the receiver (Fig. 5b). Under freely behaving conditions, the relative distance, orientation, and posture between the transmitter and receiver vary continuously as a result of locomotion, rotation, and exploratory behaviors. To assess power delivery robustness in this context, the DC output voltage was recorded in real time using an oscilloscope. Simultaneously, animal behavior was video-recorded using a camera mounted above the cage. Behavioral recordings were temporally synchronized with voltage measurements to enable correlation between behavioral states and output voltage fluctuations (Movie S2). The total recording duration was 6 min.

Observed behaviors were classified into relatively static behaviors, including resting, sniffing, and feeding, and dynamic behaviors, such as moving, rearing, and digging (Fig. 5c). Despite frequent transitions between these behavioral states, the output voltage remained near the regulated operating voltage (~9.7 V) for the majority of the recording period (Fig. 5c). When the experiments shown in Fig. 5c were repeated in four independent freely behaving trials, the temporal proportion of stable voltage operation and transient voltage-drop periods was additionally analyzed using time-averaged voltage values. Across the total 6 min freely behaving sessions, the proportion of time during which the system maintained stable voltage operation ranged from ~73.8% to 93.3% depending on the individual trial (Fig. S14).

Transient voltage reductions were occasionally observed during abrupt posture and orientation changes, particularly under large head-tilt conditions that can increase polarization mismatch between the transmitter and receiver antennas. However, these fluctuations were generally short in duration and rapidly recovered following restoration of radiative RF power. Importantly, temporary voltage drops below the nominal regulated voltage did not completely interrupt stimulation delivery. Instead, the stimulation current was transiently reduced during these events while electrical output was still maintained.

To further evaluate whether transient voltage fluctuations observed during freely behaving conditions could affect effective tDCS delivery, additional Sim4Life-based current-density simulations were performed under the experimental conditions presented in Fig. 5c (Fig. S15). A representative reference current-density threshold of 43 μA mm^−2^, based on previously reported HD-tDCS conditions, was used for comparative analysis^50^. The simulations were conducted using the maximum and minimum 1-second averaged output voltages measured during freely behaving operation, corresponding to 9.7 V and 5.65 V, respectively.

Under the nominal 9.7 V condition, the cortical area exhibiting current densities greater than the selected reference threshold of 43 μA mm^−^^2^ was ~0.656 mm^2^. Under the reduced-voltage condition (5.65 V), the cortical area exceeding the same reference threshold decreased to ~0.452 mm^2^, corresponding to ~68% of the area observed under the nominal voltage condition. The maximum depth of the cortical region exceeding the selected reference current-density threshold was also quantified. In the simulation coordinate system, where the top surface of the skull was defined as z = 0, the threshold-exceeding region extended to *z* = −0.697 mm under the nominal voltage condition and to *z* = −0.557 mm under the reduced-voltage condition. Considering the modeled skull thickness of 0.2 mm, these values correspond to cortical depths of ~0.497 mm and 0.357 mm from the brain surface, respectively. According to the Paxinos and Franklin mouse brain atlas, both depths lie within the anatomical depth range of the targeted M2 cortex^44^. This threshold-based analysis provides a comparative assessment of the spatial extent of cortical regions exceeding the selected reference current-density threshold under different output-voltage conditions. Consistent with the reduced output voltage, both the magnitude and spatial extent of the current density decreased; however, the threshold-exceeding region remained within the targeted M2 cortical region even under the reduced-voltage condition.

To experimentally validate the simulation results under realistic electrode-loading conditions, the delivered stimulation current was measured using the skull–brain phantom (Fig. S16). The phantom consisted of a 100 kΩ skull-mimicking resistor (*R*_skull_), selected based on the equivalent resistance estimated from the electrical parameters used in the previous current-density simulations, and a 0.9% agarose gel prepared in 1× PBS to emulate the conductive properties of the brain tissue. During repeated RF power ON/OFF operation, the delivered current through the skull–brain phantom was continuously recorded using a SourceMeter connected in series with the phantom load, while the RF output voltage was simultaneously monitored in real time using a digital multimeter connected to the system through soldered lead wires. The measured current profile closely followed the repeated RF power ON/OFF operation throughout the experiment, demonstrating stable and reproducible current delivery under the skull–brain phantom loading conditions (Fig. S17).

During the RF ON state, the RF output voltage was continuously maintained at the maximum regulated output voltage of ~9.7 V, while the delivered current reached ~75 μA. Based on the exposed anode area, this corresponds to a nominal electrode current density of ~780 μA mm^−2^. Although the absolute current is lower than that commonly used in previous rodent tDCS studies^51^, the smaller concentric electrode (CE) geometry enables more focal cortical stimulation, as demonstrated in our previous study^17^.

When the RF power was switched OFF, both the output voltage and delivered current rapidly decreased to the baseline level, indicating that current delivery was directly synchronized with the wireless power transmission. These results demonstrate that the proposed wireless stimulation system is capable of delivering stimulation current under the realistic electrode-loading conditions represented by the skull–brain phantom. The phantom measurement complements the current-density simulations by providing direct measurements of the delivered current under realistic electrical loading conditions, thereby offering experimental evidence of the system’s current-delivery performance.

Additional measurements were performed under repeated RF activation conditions to characterize the transient regulation behavior of the proposed wireless stimulation system (Fig. S18). The temporal response of the regulated output voltage was analyzed following RF-power onset to quantify the stabilization characteristics under practical operating conditions. The results showed that the regulated output voltage reached the target operating level (~9.7 V) within ~0.2–0.3 s after RF activation under typical experimental conditions. In addition, the transient voltage fluctuations observed during freely behaving operation were generally short in duration and rapidly recovered to the regulated voltage level.

To investigate a potential mitigation strategy for transient voltage reductions, transient measurements were also performed using an enlarged output capacitor implemented by adding a 220 µF capacitor to the original regulator output stage consisting of five 4.7 µF capacitors (Fig. S18). Under this condition, the regulated output voltage exhibited substantially slower decay behavior following transient RF-power interruption events due to the increased RC discharge time constant of the output stage. As a result, temporary voltage reductions associated with brief RF-power interruptions were significantly mitigated, leading to prolonged voltage retention during transient power-loss conditions. However, the increased output capacitance also introduced a slower transient response and longer charging time during RF-power recovery. These results indicate a design trade-off between transient voltage retention and response speed and suggest that optimization of the output-stage capacitance could further improve robustness against posture- and orientation-induced RF-power fluctuations during freely behaving operation.

To further evaluate extended-duration electrical operation under continuous radiative RF powering conditions, an additional long-term measurement was performed with the receiver module fixed under a stable RF-powering condition without animal attachment (Fig. S19). The regulated output voltage was continuously monitored over a 24 h period while RF power was continuously transmitted from the transmitter antenna. The measured output voltage remained near the target operating voltage (~9.7 V) throughout the measurement duration without noticeable interruption of electrical operation. Although this experiment does not establish chronic in vivo reliability or long-term electrode–tissue interface stability, the results provide initial evidence that the proposed RF WPT power-management architecture can sustain extended-duration electrical operation under continuous wireless powering conditions.

Collectively, these results demonstrate that the proposed radiative RF WPT–based system can sustain wireless neurostimulation under continuously changing freely behaving conditions while maintaining rapid voltage recovery characteristics against transient RF-power fluctuations. Although temporary voltage drops were occasionally observed during abrupt posture and orientation changes, the regulated output voltage recovered rapidly after RF-power restoration. The threshold-based current-density simulation further indicated that a portion of the target cortical area remained above the selected reference current-density threshold under the reduced-voltage condition. In addition, the enlarged output capacitance exhibited prolonged voltage retention characteristics, suggesting a potential strategy for further improving transient robustness under practical freely behaving operation. Together, these findings support the feasibility of the proposed system for battery-free wireless neurostimulation experiments in freely behaving animals without reliance on tethered connections or constrained experimental infrastructure.

### Behavioral validation of wireless tDCS via M2 stimulation

We employed a FPCB-based CE, previously validated in our earlier work^17^, for tDCS using the fully integrated wireless system (Fig. S20). In all configurations, the anode was modeled as a circular electrode with a diameter of 0.35 mm, and the distance between the anode and cathode was fixed at 0.125 mm. For the CE-tDCS configuration, the cathode was designed as a concentric ring surrounding the central anode, with inner and outer radii of 0.3 mm and 0.5 mm, respectively. The electrode layout was further designed to enable selective stimulation of the left or right secondary motor cortex (M2) by positioning a reference hole at the bregma, allowing precise hemispheric targeting.

Prior to neuromodulation experiments, we first assessed whether attachment of the radiative RF WPT–based tDCS system affected spontaneous mouse behavior. The complete system comprised a 0.9 g receiver module, a 0.92 g crown structure, and a 0.008 g tDCS electrode (Figs. S21 and  S22). When all components were fully integrated, the final total weight of the system was ~1.8 g, corresponding to ~7% of the body weight of the mice used in this study (Figs. S21 and  S22). This load falls within the range generally considered unlikely to induce significant behavioral interference^52–54^. Although the present receiver architecture remains larger than some previously reported ultra-miniaturized implantable wireless neural interfaces, the current system integrates a radiative RF receiving antenna, RF–to–DC rectification circuitry, DC–DC power management, and stimulation electronics within a fully battery-free platform capable of cage-scale wireless operation. This architecture therefore represents a practical trade-off between miniaturization and robust radiative RF wireless power transfer under freely behaving experimental conditions.

To quantitatively evaluate potential behavioral effects, OFTs were conducted both before surgery and after system implantation^55,56^ (Movie S3). The surgical procedures were performed as follows (Fig. S23). The same animals were evaluated under identical experimental conditions both in the pre-implantation state and after attachment of the radiative RF WPT-based tDCS system. All mice were allowed a minimum recovery and habituation period of one week after surgery before behavioral testing. Analysis revealed no significant differences in movement trajectories, total travel distance, or average locomotion speed between pre- and post-implantation conditions (Figs. S24 and  S25). In addition, to evaluate the potential behavioral effects of continuous RF power transmission, OFT experiments were performed under sequential RF OFF and RF ON conditions using the wireless system without tDCS electrode attachment (Fig. S26 and Movie S4). During these experiments, the transmitter continuously delivered 4 W RF power, corresponding to the maximum RF input condition evaluated in this study. Comparative analysis revealed no significant differences in total traveling distance or overall locomotor behavior between RF OFF and RF ON conditions across four independent animals (Fig. S26). Representative movement trajectories further demonstrated comparable exploratory behavior during RF exposure, suggesting that continuous 2.45 GHz RF transmission under the applied experimental conditions did not produce measurable behavioral changes during the experimental session. These findings indicate that neither the system weight, head-mounted configuration, nor continuous RF power transmission substantially affects spontaneous locomotor behavior.

Following this validation, in vivo experiments were performed to evaluate the neuromodulatory efficacy of the proposed system. The secondary motor cortex (M2), which plays a critical role in motor planning and directional movement control, was selected as the stimulation target. Experiments were conducted under radiative RF WPT conditions, with the transmitting antenna positioned above the cage (Fig. S27). Electrical stimulation was delivered wirelessly to the anode implanted over the right M2 region via the tDCS electrodes.

**Fig. 6:**
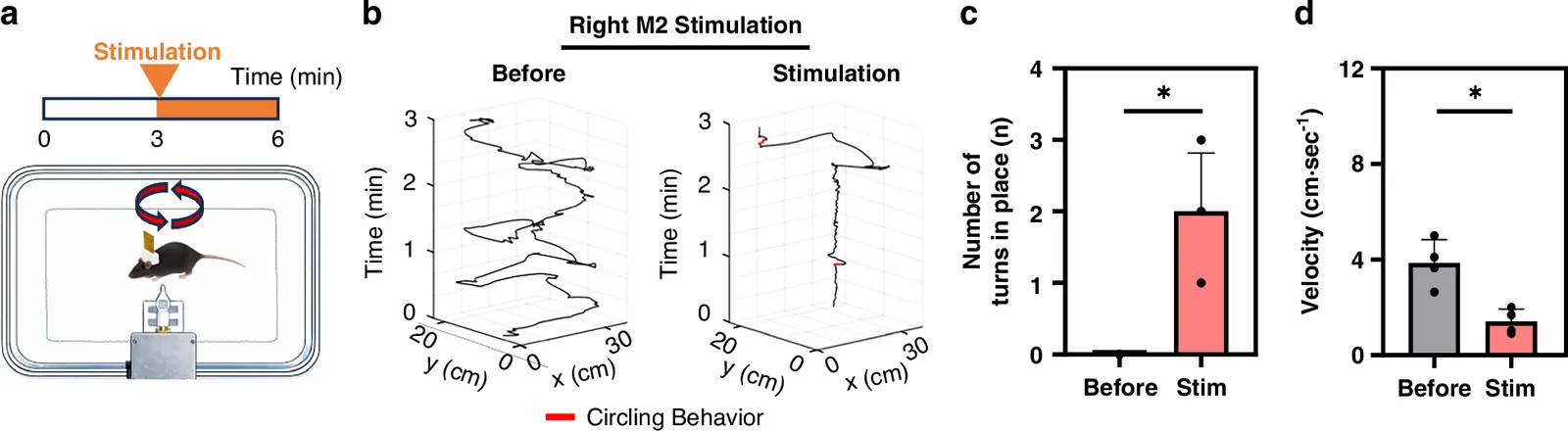
**Behavioral validation of wireless tDCS via right M2 stimulation in freely behaving mice.** **a** Schematic of the stimulation paradigm (3 min OFF, 3 min ON) used to assess stimulation-induced counterclockwise rotation following right secondary motor cortex (M2) tDCS. **b** Time-resolved 3D trajectories showing rotational behavior before and during stimulation. **c** Quantification of in-place rotations before and during stimulation. **d** Comparison of locomotor velocity before and during stimulation. Data are presented as mean ± SD (*n* = 4 independent animals). Two-tailed paired Student’s *t* tests were performed (*p < 0.05): **c** *t*(3) = 4.899, *p* = 0.0163; **d** *t*(3) = 4.373, *p* = 0.0221

Each experimental session lasted 6 min and consisted of a 3-min baseline (OFF) period without stimulation followed by a 3-min stimulation (ON) period (Fig. 6a). Mouse behavior was continuously recorded using an overhead camera throughout the session.

During the baseline period, mice freely explored the cage and exhibited broad locomotor trajectories. In contrast, during M2 stimulation, mice showed a reduction in movement range and average locomotion speed (Figs. 6b and  S28 and Movie S5). Notably, the frequency of repetitive 360° rotations about the vertical body axis, defined as circling behavior, significantly increased during the stimulation period (Fig. 6c). The overall average locomotion speed was markedly reduced compared with the pre-stimulation condition (Fig. 6d). Trajectory analysis revealed a clear transition from widespread exploratory movement during baseline to localized circular motion centered around a fixed rotation point during stimulation. This behavioral phenotype is consistent with previously reported effects of electrical stimulation targeting the M2 region^57,58^.

Together, these results demonstrate that the proposed radiative RF WPT–based tDCS system not only delivers stable power under radiative conditions but also enables functionally effective neuromodulation capable of eliciting robust, target-specific behavioral outcomes in freely behaving mice. This study thus establishes the feasibility of a fully wireless, battery-free tDCS neurostimulator for causal neuromodulation and behavioral validation under freely behaving in vivo conditions with reduced physical constraints.

## Conclusion

In this study, we propose and experimentally validate a fully wireless tDCS neurostimulator based on 2.45 GHz radiative RF WPT for operation in freely behaving animal models. A key feature of the proposed system is its ability to support wireless power delivery and effective neuromodulation at the cage scale without relying on onboard batteries or coil-embedded experimental infrastructures commonly required in near-field WPT systems.

To address the longstanding challenges of posture sensitivity and alignment dependence associated with radiative RF WPT, we develop an FPCB–based monopole antenna with quasi-omnidirectional radiation characteristics and optimize both antenna behavior and system-level integration. In addition, integration of an RF–to–DC rectifier and a DC–DC converter within an integrated power management architecture enables the system to maintain a regulated high-voltage output of ~9.7 V over practical ranges of distance and orientation encountered during freely behaving conditions.

Experiments in freely behaving mice demonstrated that the proposed system maintained the regulated operating voltage for the majority of the experimental period across diverse behavioral states, although transient voltage reductions were occasionally observed during abrupt posture and orientation changes, particularly under large head-tilt conditions that can increase polarization mismatch between the transmitter and receiver antennas. However, these fluctuations were generally short in duration and rapidly recovered following restoration of the received RF power. These results indicate that radiative RF WPT can support wireless neurostimulation in freely behaving in vivo environments with substantially reduced tethering and environmental constraints. Furthermore, targeted tDCS of the secondary motor cortex (M2) consistently induces circling behavior, providing behavioral-level evidence that wirelessly delivered power produces functionally meaningful neuromodulatory effects.

Unlike prior radiative RF WPT–based bioelectronic studies that primarily emphasize output voltage or energy conversion efficiency, this work presents an integrated in vivo validation covering wireless power delivery, stimulation circuit operation, neural modulation, and behavioral outcomes under freely behaving conditions. These findings challenge the prevailing view that radiative RF WPT is inherently unsuitable for freely behaving animal experiments.

Although the present receiver architecture still imposes partial physical constraints due to the size and footprint of the head-mounted module and the vertically oriented antenna structure required for stable radiative RF power reception, the proposed system substantially reduces the behavioral restrictions associated with conventional tethered or coil-embedded wireless stimulation platforms. In the current implementation, the integrated receiver module and supporting crown structure have a total weight of ~1.8 g, and stable operation requires an overhead transmitting antenna positioned above the behavioral arena. Nevertheless, open field experiments demonstrated that the mounted system did not produce significant alterations in spontaneous locomotor behavior under the tested conditions.

Importantly, the proposed architecture is inherently extensible along multiple technical dimensions. First, by incorporating a diplexer-based front-end that enables simultaneous utilization of 2.45 and 5.8 GHz ISM bands, the system can support frequency-division–based multi-site stimulation or parallel power routing within a single compact receiver module. Such multiband operation provides a pathway toward independent control of spatially separated stimulation nodes without increasing mechanical complexity or adding additional wired connections.

Second, further antenna miniaturization can be achieved by shifting the operating frequency to higher microwave bands, such as the 24-GHz ISM band. The reduced wavelength at 24 GHz enables a substantial reduction in the footprint of the receiving antenna, facilitating further miniaturization of future head-mounted receiver architectures. Such miniaturization can significantly reduce the physical burden on the animal and enable experimental paradigms involving multiple freely behaving subjects, such as social interaction studies, where each animal is powered wirelessly without tethering.

Third, future versions of the proposed platform could incorporate low-power programmable constant-current stimulation architectures to enable dynamic control of stimulation parameters, including stimulation amplitude, duration, duty cycle, and delivered dose. In the present study, a voltage-driven stimulation architecture was intentionally selected because it enables lower power consumption and reduced circuit complexity compared with fully programmable current-controlled stimulation systems, thereby improving compatibility with the limited harvested power budget of the battery-free radiative RF WPT platform. However, under voltage-driven operation, variations in delivered stimulation current may arise due to changes in electrode–tissue interface impedance, contact conditions, and inter-animal biological variability. To address these limitations, ultra-low-power feedback-based current regulation circuits using micropower operational amplifiers and low-power microcontrollers may enable programmable stimulation control while maintaining compatibility with the harvested RF power budget of the present radiative RF WPT platform. In addition, integration of impedance-monitoring or feedback-assisted regulation schemes may further improve stimulation reproducibility, dose controllability, and long-term operational stability under freely behaving conditions.

Furthermore, the present study is currently limited to skull-surface tDCS neuromodulation and short-term behavioral validation in freely behaving animals. Future studies incorporating chronic operation, long-term behavioral assessment, and integration with fully implantable neural interface systems may further expand the applicability and translational potential of the proposed radiative RF WPT platform.

Overall, the proposed radiative RF WPT–based tDCS system represents a battery-free platform that demonstrates stable extended-duration electrical operation under continuous wireless powering conditions. This work establishes the feasibility of radiative RF wireless power transfer for behavioral neuromodulation in freely behaving animals and provides a technological foundation for future battery-free neural interfaces, multiband RF energy harvesting, and next-generation wireless neurostimulation platforms.

## Supplementary information

Supporting Information

Thermal imaging recording with the RF transmitter turned off and on

Output voltage measurement of the radiative RF WPT neurostimulator during freely behaving conditions

Open-field test recordings of a mouse before and after mounting the radiative RF WPT neurostimulation system

Open-field test before and during RF power transmission in freely behaving mice

Representative video showing mouse behavior during baseline and during right M2 stimulation
