# Supplementary material for: Battery-free, fully wireless neurostimulation via radiative RF power transfer in freely behaving mice
Source: Microsyst Nanoeng. 2026 Sep 28;12:332. doi: 10.1038/s41378-026-01439-2 (PMC13620167; doi:10.1038/s41378-026-01439-2)
Supplement: Supplementary file 1 [file 41378_2026_1439_MOESM1_ESM.docx]

Supporting Information for

**Battery-Free, Fully Wireless Neurostimulation**

**via Radiative RF Power Transfer in Freely Behaving Mice**

Kyungbin Yoo^a,†^, Sehwan Park^a,†^, Jimin Yang^a^, Minseok Kim^a^, Seungjun Lee^a,b^, Giheon Kim^a^, Namsun Chou^b,c^, Hyogeun Shin^a,d,e,^*, Sehoon Park^a,d,^*

^a^School of Electronic and Electrical Engineering, Kyungpook National University, Daegu, Republic of Korea

^b^Emotion, Cognition & Behavior Research Group, Korea Brain Research Institute (KBRI), Daegu, Republic of Korea

^c^Graduate School, Daegu Gyeongbuk Institute of Science and Technology (DGIST)

Daegu, Republic of Korea

^d^School of Electronics Engineering, Kyungpook National University, Daegu, Republic of Korea

^e^Brain Science and Engineering Institute, Kyungpook National University, Daegu, Republic of Korea

† These authors contributed equally to this work.

*Corresponding authors:

Hyogeun Shin ([hyogeunshin@knu.ac.kr](mailto:hyogeunshin@knu.ac.kr))

Sehoon Park ([sehoon.park@knu.ac.kr](mailto:sehoon.park@knu.ac.kr))

Lead contact: Sehoon Park

**Supplementary Figures**

**Fig. S1. Cross-sectional schematic of the double-sided FPCB structure.** The stack consists of symmetric copper layers separated by polyimide and adhesive films, with a bottom stiffener to enhance mechanical rigidity. The total thickness of the structure is 364 μm.

**Fig. S2. Measurement-Based I–V Characterization of the Zener Diode for Nonlinear Modeling.** The modeling was based on the experimentally measured DC voltage across the Zener diode and the DC current flowing through the Zener diode.

**Fig. S3. Measurement-based steady-state equivalent input resistance of the DC–DC converter (kΩ).** The equivalent resistance is calculated from the measured output voltage of Voltage Doubler and estimated input current of DC-DC Converter.

**Fig. S4. Simulated antenna gain (dBi) on (a) Standalone 50 Ω matched antenna and (b) Antenna on fully integrated system at CST.** The radiation patterns and gain characteristics are highly similar.

**Fig. S5. SAR simulation environment implemented in CST Microwave Studio under the worst-case transmitter-to-receiver configuration (separation < 1 mm).** The simulation consisted of a transmitting antenna, a head-mounted receiving antenna, and a homogeneous dielectric mouse model. The transmitting antenna was positioned above the mouse using the same experimental geometry employed during the freely behaving experiments. The mouse model was assigned equivalent biological tissue properties with a relative permittivity (ε_r_) of 44.8 and an electrical conductivity (σ) of 2.1 S m^-1^. Peak spatial-average SAR was calculated using the IEEE/IEC 62704-1–compliant 0.1 g spatial-averaging algorithm.

**Fig. S6. Simulated antenna gain of laid receiver module in linear on mouse model (ε = 45) with crown at 2.45 and 5.8 GHz.** Polarization mismatch occurs due to misalignment between the transmitter and receiver.

**Fig. S7. Simulated radiation intensity distributions of the proposed antenna observed from six principal viewing directions (top, bottom, front, back, left, and right) at 2.45 and 5.8 GHz, shown in free space and when mounted on a mouse model (ε = 45).** *Gain_Norm.dB_: Normalized gain, where Gain_Norm.dB_ = Gain/Gain_Max.dB_, Gain_Max.dB_ = 4.09 dBi

**Fig. S8. Simulated and measured reflection coefficients (S11) of the 50 Ω matched antenna.** The 50 Ω configuration was employed to evaluate the intrinsic resonant and radiation characteristics of the proposed antenna structure independent of system-level loading effects.

**Fig. S9. RF-Power transfer experiment setup in an acrylic box (40 × 40 × 15 cm³).** The experimental setup consisted of a signal generator operating at 2.45 GHz, a power amplifier and its supporting stand, a dual-band transmitter antenna operating at 2.45 and 5.8 GHz, an acrylic box, and a multimeter for measuring the output voltage.

**Fig. S10. Measured output voltage inside an 80 × 80 × 15 cm³ acrylic enclosure under 4 W RF transmission with a 100 kΩ load resistance (φ = 90°*,* θ *=* 0°).**

**Fig. S11. Measured output voltage in a (40 × 40 × 15 cm³) acrylic enclosure under 2 W RF transmission as a function of φ (0°, 90°, 180 °, 270°) and θ (0°, 45°) with a 100 kΩ load resistance.**

**Fig. S12. Simulated radiation patterns of the antenna on fully integrated system at 2.45 GHz, obtained using HFSS with a feed structure geometrically approximated to the implemented configuration.** The linear gain was evaluated at a fixed elevation angle of θ = 18° while sweeping the azimuthal angle φ. Results are shown for configurations without DC leads and with DC leads to assess the influence of DC wiring on angular radiation characteristics.

**Fig. S13. Temperature measurements of four different mice with the RF transmitter turned on and off.** Scalp temperature was monitored for 10 min, and the data were sampled every 6 s. The averaged temperature variation across the four mice during the 10 min measurement period was approximately 0.03 °C. *∆T_AVG_ = T_RF–On–AVG_-T_RF–Off–AVG_, where T_RF–On–AVG_ and T_RF–Off–AVG_ denote the averaged scalp temperatures measured during RF–on and RF–off conditions, respectively.

**Fig. S14. Schematic illustration of the voltage-analysis method used to quantify stable-voltage and transient voltage-drop periods during freely behaving wireless operation.** The “Stable-voltage region” was defined as the period during which the output voltage continuously remained near the nominal operating voltage (~9 V), whereas the “Voltage-drop region” corresponded to transient intervals in which the output voltage decreased below the nominal level before recovering back to ~9 V. The temporal proportion of each region was used to calculate the percentage of stable-voltage maintenance and transient voltage-drop occurrence during the stimulation sessions.

**Fig. S15. Current-density simulations under freely behaving wireless tDCS operating conditions.** Current-density distributions within cortical tissue were analyzed under the maximum (9.7 V) and minimum (5.65 V) 1-second averaged output voltage conditions measured during freely behaving operation. The red contour indicates the reference current-density threshold of 43 μA mm^-^² used for comparative spatial evaluation. The cortical area exceeding the selected reference current-density threshold decreased from approximately 0.656 mm² at 9.7 V to 0.452 mm² at 5.65 V, corresponding to approximately 68% of the area observed under the nominal voltage condition. The white labels indicate the maximum depth of the region exceeding the selected reference current-density threshold in the simulation coordinate system, measured from the skull surface (*z* = 0), which reached 0.697 mm and 0.557 mm under the 9.7 V and 5.65 V conditions, respectively. This threshold-based analysis compares the spatial extent and maximum penetration depth of cortical regions exceeding the selected reference current-density threshold under different output-voltage conditions.

**Fig. S16. Experimental setup for delivered-current measurement using a skull–brain phantom that emulates realistic electrode-loading conditions.** The phantom comprised a 100 kΩ skull-mimicking resistor (R_skull_) and a 0.9% agarose gel prepared in 1× PBS, representing the skull and brain tissue, respectively. The proposed wireless stimulation system was operated under repeated RF power ON/OFF conditions during the current-measurement experiment.

**Fig. S17. Results of delivered-current measurement using a skull–brain phantom that emulates realistic electrode-loading conditions.** The current was measured using a skull–brain phantom consisting of a 100 kΩ skull-mimicking resistor (R_skull_) and a 0.9% agarose gel prepared in 1× PBS to emulate the electrical properties of the brain tissue. The measured current profile was recorded during repeated RF power ON/OFF operation. During the RF ON state, corresponding to the maximum regulated output voltage of the proposed system (9.7 V), the delivered current reached approximately 75 μA, whereas the current returned to the baseline level during the RF OFF state.

**Fig. S18. Transient output-voltage behavior of the proposed radiative RF WPT system under intermittent RF powering conditions at Tx–Rx separation distances (D_TX-RX_) of 10 cm and 22 cm. Panels (a,b) show the responses without a large load capacitor, while panels (c,d) show the responses with a large load capacitor connected at the output.** T_10cm_, T_22cm_, T_10cm–cap_, T_22cm–cap_ denote the regulation recovery time required for the output voltage to recover to 95% of the regulated voltage (∼9.7 V) after RF reactivation. Multiple recovery events with different residual capacitor voltages are presented to evaluate the transient response under varying discharge conditions. In **(a)**, the measured T_10cm_ values were 0.31 s, 0.19 s, 0.23 s, and 0.27 s in chronological order. In **(b)**, the measured T_22cm_ values were 0.28 s, 0.23 s, 0.28 s, and 0.22 s, respectively. With the addition of a large load capacitor, the recovery times increased due to the larger charging requirement. The measured T_10cm–cap_ values in **(c)** were 2.57 s, 1.28 s, and 1.6 s, whereas the measured T_22cm–cap_ values in **(d)** were 8 s, 3.3 s, and 5 s, respectively.

**Fig. S19. Long-term output-voltage stability measurement of the proposed radiative RF WPT system over a continuous 24 h operation period under a fixed Tx–Rx configuration.** The receiver was mechanically fixed at a Tx–Rx separation distance of 22 cm while 4 W RF power was continuously transmitted from the transmitter. The output voltage was sampled at 1-second intervals throughout the experiment. Under this controlled stationary condition without movement-induced alignment variation, the measured output voltage remained consistently near the regulated voltage of ∼9.7 V with minimal fluctuation over the entire 24 h period, demonstrating stable extended-duration electrical operation and continuous wireless power delivery capability of the proposed system.

**Fig. S20. Layout and microscopic image of the flexible concentric electrode (CE-tDCS).** The electrode was configured in a concentric electrode (CE) geometry for CE-tDCS and designed to selectively target either the left or right secondary motor cortex (M2). The reference holes exhibit a bilaterally symmetric structure, enabling precise alignment with bregma during surgical implantation. This design ensures accurate positioning of the electrode over the intended left or right M2 cortical region.

**Fig. S21. Weight distribution of the radiative RF WPT-based system components.**
Individual weights of each component constituting the radiative RF wireless power transfer (WPT)-based neurostimulator—including the crown, receiver module, and tDCS electrode—are presented, along with the total weight of the fully integrated system.

**Fig. S22. Components and head-mounted configuration of the radiative RF WPT- based system.** The system consists of a main receiver module, an FPCB-based tDCS electrode, and a custom-designed 3D-printed crown for stable cranial fixation. A representative photograph of a mouse with the fully assembled device mounted on the skull demonstrates the compact and lightweight architecture, optimized for freely moving rodent experiments.

**Fig. S23. Surgical procedure for implanting the radiative RF wireless power transfer tDCS system. (a)** The mouse was anesthetized and secured in a stereotaxic frame, and the scalp was removed to expose the skull. **(b)** For effective stimulation, the skull over the target region, the secondary motor cortex (M2), was carefully thinned. **(c)** A stainless steel anchoring screw was fixed to provide additional mechanical stability, and the concentric electrode was positioned over the target cortical region (M2). **(d)** A protective crown was mounted to stably support the stimulation module, and the entire assembly was secured using dental cement.

**Fig. S24. Locomotor trajectory maps of mice under freely moving conditions before and after system mounting.** Locomotor trajectories from Mouse 1, Mouse 2 and Mouse 3 during a 20-minute open-field test, pre- and post-mounting. Velocity is color-coded.

**Fig. S25. Tracking analysis of locomotor activity before and after system mounting. (a)** Time-resolved distance traveled by Mice 1-3, shown in 5-min intervals. Black cirecles and solid lines represent pre-mounting data; red squares and dashed lines represent post-mounting data. **(b)** Corresponding velocity profiles of Mice 1-3 over same intervals. **(c)** Distances traveled during each 5-min interval. **(d)** Total distance traveled and **(e)** mean velocity over the 20-min session. Data are presented as the mean ± standard deviation (*n* = 3 mice). Statistical analysis was performed using two-tailed paired Student’s t-tests. ns, not significant. **(c)** t(2) = 0.1780, *p* = 0.8751 (5 min), t(2) = 1.022, *p* = 0.4142 (10 min), t(2) = 2.370, *p* = 0.1412 (15 min), and t(2) = 0.6393, *p* = 0.5881 (20 min) for each 5-min interval; **(d)** t(2) = 1.274, *p* = 0.3308; **(e)** t(2) = 1.274, *p* = 0.3308.

**Fig. S26. Evaluation of the behavioral effects of RF power transmission during open field test (OFT) in freely behaving mice. (a)** Schematic illustration of the experimental protocol comparing	g RF OFF and RF ON conditions to assess whether RF power exposure affects mouse behavior. **(b)** Representative image of the OFT setup, consisting of a wireless power transmitter placed beneath the arena and a mouse implanted with the wireless system without tDCS electrode attachment. **(c)** Quantitative analysis of total traveling distance under RF OFF and RF ON conditions. Data are presented as mean ± SD (n = 4 mice). Two-tailed paired Student’s t-tests were performed. ns, not significant. t(3) = 0.4177, p = 0.7043**. (d)** Movement trajectories of four individual mice under RF OFF (blue) and RF ON (orange) conditions

**Fig. S27. Experimental setup for stimulation-induced rotational behavior assessment.** A behavioral cage (38 × 23 × 19.5 cm³) was used for the experiment, with a 2.45 GHz RF transmitter positioned at the center of the cage to deliver wireless power. Left: Photograph of the overall experimental setup. Right: Top-view image of the behavioral arena highlighting the transmitter placement.

**Fig. S28. Time-resolved 3D trajectories colored by velocity during right M2 Stimulation.** 3D locomotor trajectories before and after stimulation of the right M2 region. Velocity is color-coded. **Supplementary Tables**

**Table S1. Summary of system specifications and comparison with existing RF-based wireless power transfer neurostimulator systems for small animals.**

|  | **This work** | **[1]** | **[2]** | **[3]** | **[4]** | **[5]** |
| --- | --- | --- | --- | --- | --- | --- |
| **Mechanism** | **Radiative RF** | **Inductive coupling** | **Inductive coupling** | **Inductive coupling** | **Inductive coupling** | **Inductive coupling** |
| **Resonance frequency** | **2.45 GHz** | **13.56 MHz** | **13.56 MHz** | **898 MHz** | **1.87 / 16.3 MHz** | **13.56 MHz** |
| **Customized experimental arena required** | **No** | **Yes**  **(Coil integrated)** | **Yes**  **(Coil integrated)** | **No** | **No** | **Yes**  **(Coil integrated)** |
| **Active power transfer range^†^**  **(cm)** | **~ 40** | **Entire arena volume** | **Entire arena volume** | **< 1** | **< 1** | **Entire arena volume** |
| **Experimental Arena**  **(cm^3^)** | **38 x 28 x 19.5** | **46 x 24 x 20** | **28 x 22 x N/A** | **33 x 33 x 42** | **-** | **Diameter = 20, Height = N/A** |
| **Tx structure** | **1 x Meandered monopole antenna** | **1 x Tx coil ^*^**  **4 x resonantor coil ^*^** | **1 x Tx coil ^*^** | **1 x Tx coil**  **1 x resonantor coil** | **1 x Tx coil** | **1 x Tx coil** |
| **Rx weight**  **(g)** | **1.65** | **5.7** | **0.046** | **N/A** | **0.451** | **0.2** |
| **Rx dimensions (mm^2^)** | **38 x 21.2** | **19 x 19** | **0.5 x 0.19** | **d_outR_= 0.5** | **9 x 13** | **10 x 10** |

† Active power transfer range is defined as the effective power transfer region. For inductive systems employing cage-integrated resonant coils, the effective range corresponds to the spatial extent of the resonant enclosure. For RF radiative systems using multiple transmitting antennas, power delivery is designed to provide distributed coverage across the experimental arena rather than a discrete Tx–Rx separation distance.

**Table S2. Simulated antenna input impedance obtained using CST under four configurations:** (a) Standalone 50 Ω-matched reference antenna, (b) Antenna on Full system in free space and (c) Antenna on Full system mounted on a dummy mouse model (ε = 2.7)

| **Simulation conditions** | **Input Impedance (Unit = (Ω))** |
| --- | --- |
| **(a) Standalone 50 Ω matched in Air** | **45.7 + j20** |
| **(b) Antenna on Full system in Air** | **71.3 + j11.5** |
| **(c) Antenna on Full system on dummy mouse** | **58 – j4** |

**Table S3. Summary of the link budget parameters employed for estimating the received RF power at 2.45 GHz**. (P_SG_ = Power of Signal Generator, L_cable_ = Loss of RF transmission cable, G_PA_ = Gain of Power Amplifier, G_t_ = Transmitter Antenna Gain, L_FSPL_ = Free space path loss calculated using Friis equation, G_r_ = Receiver Antenna Gain)

| **Link Budget parameters** | **Data** |
| --- | --- |
| **P_SG_** | **3~19 dBm** |
| **L_Cable_** | **~ 0.75 dB** |
| **G_PA_** | **18 ± 1 dB** |
| **G_t_** | **~ 0.5 dBi (Measured)** |
| **L_FSPL_** | **~ 19.3 dB (at 9 cm, 2.45 GHz)** |
| **G_r_** | **~1.76 dBi (Simulated, at φ = 90°, θ = 0°)** |

**Table S4. Summary of CST-simulated peak gain of 50 Ω matched antenna under free-space, biological loading and mechanical deformation conditions at 2.45 and 5.8 GHz : Peak Gain of Figures. 2c~2e.**

| **Frequency** | **Peak Gain**  **(Fig. 2c. Antenna only)** | **Peak Gain**  **(Fig. 2d. On the mouse model)** | **Peak Gain**  **(Fig. 2e. Bending 22.5°)** | **Peak Gain**  **(Fig. 2e. Bending 45°)** |
| --- | --- | --- | --- | --- |
| **2.45 GHz** | **2.05** | **2.26** | **2.08** | **2.1** |
| **5.8 GHz** | **3.1** | **3.51** | **4.06** | **4.09** |

**Supplementary Movies**

**Movie S1. Thermal imaging recording with the RF transmitter turned off and on.**

**Movie S2. Output voltage measurement of the radiative RF WPT neurostimulator during freely behaving conditions.**

**Movie S3. Open-field test recordings of a mouse before and after mounting the radiative RF WPT neurostimulation system.**

**Movie S4. Open-field test before and during RF power transmission in freely behaving mice.**

**Movie S5. Representative video showing mouse behavior during baseline and during right M2 stimulation.**

1	Lee, B. *et al.* An inductively-powered wireless neural recording and stimulation system for freely-behaving animals. *IEEE transactions on biomedical circuits and systems* **13**, 413–424 (2019).

2	Burton, A. *et al.* Wireless, battery-free, and fully implantable electrical neurostimulation in freely moving rodents. *Microsystems & nanoengineering* **7**, 62 (2021).

3	Lee, A.-H., Lee, J., Leung, V., Larson, L. & Nurmikko, A. Patterned electrical brain stimulation by a wireless network of implantable microdevices. *Nature Communications* **15**, 10093 (2024).

4	Moon, J. *et al.* Battery‐Free Dual‐Channel Wireless Neurostimulator Using Passive Circuitry for Neuropathic Pain Modulation. *Advanced Healthcare Materials* **14**, e02443 (2025).

5	Li, J. *et al.* A wireless, battery-free device for electrical neuromodulation of bladder contractions. *Materials Today Bio* **28**, 101233 (2024).
